# Heterolytic bond activation at gold: evidence for gold(iii) H–B, H–Si complexes, H–H and H–C cleavage†
†Electronic supplementary information (ESI) available: Synthesis, NMR spectroscopic and computational details. See DOI: 10.1039/c8sc05229h


**DOI:** 10.1039/c8sc05229h

**Published:** 2019-01-16

**Authors:** Luca Rocchigiani, Peter H. M. Budzelaar, Manfred Bochmann

**Affiliations:** a School of Chemistry , University of East Anglia , Norwich Research Park , Norwich NR4 7TJ , UK . Email: l.rocchigiani@uea.ac.uk ; Email: m.bochmann@uea.ac.uk; b Department of Chemistry , University of Naples Federico II , Via Cintia , 80126 Naples , Italy . Email: p.budzelaar@unina.it

## Abstract

The coordinatively unsaturated gold(iii) chelate complex [(C^N–CH)Au(C_6_F_5_)]^+^ (**1**^+^) reacts with main group hydrides H–BPin and H–SiEt_3_ in dichloromethane solution at –70 °C to form the corresponding σ-complexes, which were spectroscopically characterized (C^N–CH = 2-(C_6_H_3_Bu^*t*^)-6-(C_6_H_4_Bu^*t*^)pyridine anion; Pin = OCMe_2_CMe_2_O). In the presence of an external base such as diethyl ether, heterolytic cleavage of the silane H–Si bond leads to the gold hydrides [{(C^N–CH)AuC_6_F_5_}_2_(μ-H)]^+^ (**2**^+^) and (C^N–CH)AuH(C_6_F_5_) (**5**), together with spectroscopically detected [Et_3_Si–OEt_2_]^+^. The activation of dihydrogen also involves heterolytic H–H bond cleavage but requires a higher temperature (–20 °C). H_2_ activation proceeds in two mechanistically distinct steps: the first leading to **2** plus [H(OEt_2_)_2_]^+^, the second to protonation of one of the C^N pyridine ligands and reductive elimination of C_6_F_5_H. By comparison, formation of gold hydrides by cleavage of suitably activated C–H bonds is very much more facile; *e.g.* the reaction of **1·OEt_2_** with Hantzsch ester is essentially instantaneous and quantitative at –30 °C. This is the first experimental observation of species involved in the initial steps of gold catalyzed hydroboration, hydrosilylation and hydrogenation and the first demonstration of the ability of organic C–H bonds to act as hydride donors towards gold.

## Abstract 2

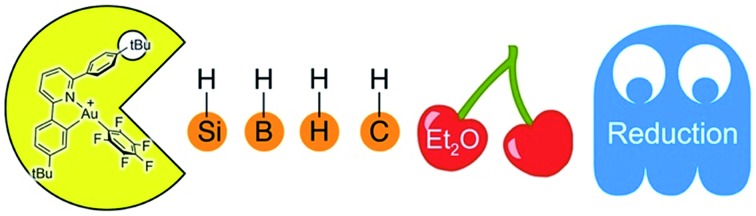
Gold(iii) forms spectroscopically detectable H–B and H–Si σ-complexes; experiments and DFT calculations demonstrate heterolytic H–Si, H–H and H–C bond cleavage.

## Introduction

Gold catalysts, both heterogeneous and homogeneous, have experienced an explosive growth in interest in recent years.^1^–^3^ In the absence of direct evidence, their mode of action tends to be explained in analogy to the well-known chemistry of other noble metals, notably palladium and platinum, and indeed gold and platinum systems are often compared side-by-side.^4^,^5^ There are significant differences, however, not least in the distinct reluctance of gold to undergo oxidative addition reactions,^6^,^7^ and an experiment-based outline of the reactivity of gold, and in particular of the much less well explored chemistry of gold(iii), is only now beginning to emerge.^8^–^10^ Heterogeneous gold catalysts show high activity in a multitude of reactions, for example in hydrogenations,^11^–^16^ including the hydrogenation of nitro compounds^17^–^19^ and hydrogen transfer,^20^ in acetylene hydrochlorination,^21^ in the water–gas shift reaction,^22^ in hydrosilylations and in catalytic dehydrogenative Si–O coupling reactions.^23^–^28^ Similarly, homogeneously gold-catalyzed hydrogenations and hydrosilylations^29^–^31^ as well as alkyne hydroborations^32^ have been reported.

Details of the reactions of H_2_, silanes and boranes with gold compounds, and particularly of their modes of activation and reaction pathways, are however rather scarce. The common feature of all these reactions is that they involve, or are postulated to involve, Au–H species. While in a number of heterogeneous systems surface gold hydride species could indeed be detected spectroscopically,^33^–^35^ the mechanisms of gold-catalysed reactions have been explored by computational modelling. Naturally, most studies are based on mechanistic analogies to better known noble metal catalysts; for example, homolytic H–H and H–Si bond scission and oxidative addition of dihydrogen or of silanes to surface gold atoms are assumed, to give Au–H and Au–SiMe_3_ species which initiate the catalytic cycles.^24^–^28^,^35^–^37^ There are however also cases where the possible involvement of polar solvents in the catalytic process has been recognized, leading to models of heterolytic, solvent-facilitated H–H bond cleavage.^38^–^41^

We present here the first experimental evidence for detectable gold(iii) adducts with borane H–B and silane H–Si bonds, elucidate the important role of the solvent and of basic ligand sites in the formation of Au–H species *via* heterolytic H–Si and H–H bond scission, and demonstrate the ability of gold(iii) to generate gold hydrides by H–C(sp^3^) bond cleavage.

## Results and discussion

**Scheme 1:**
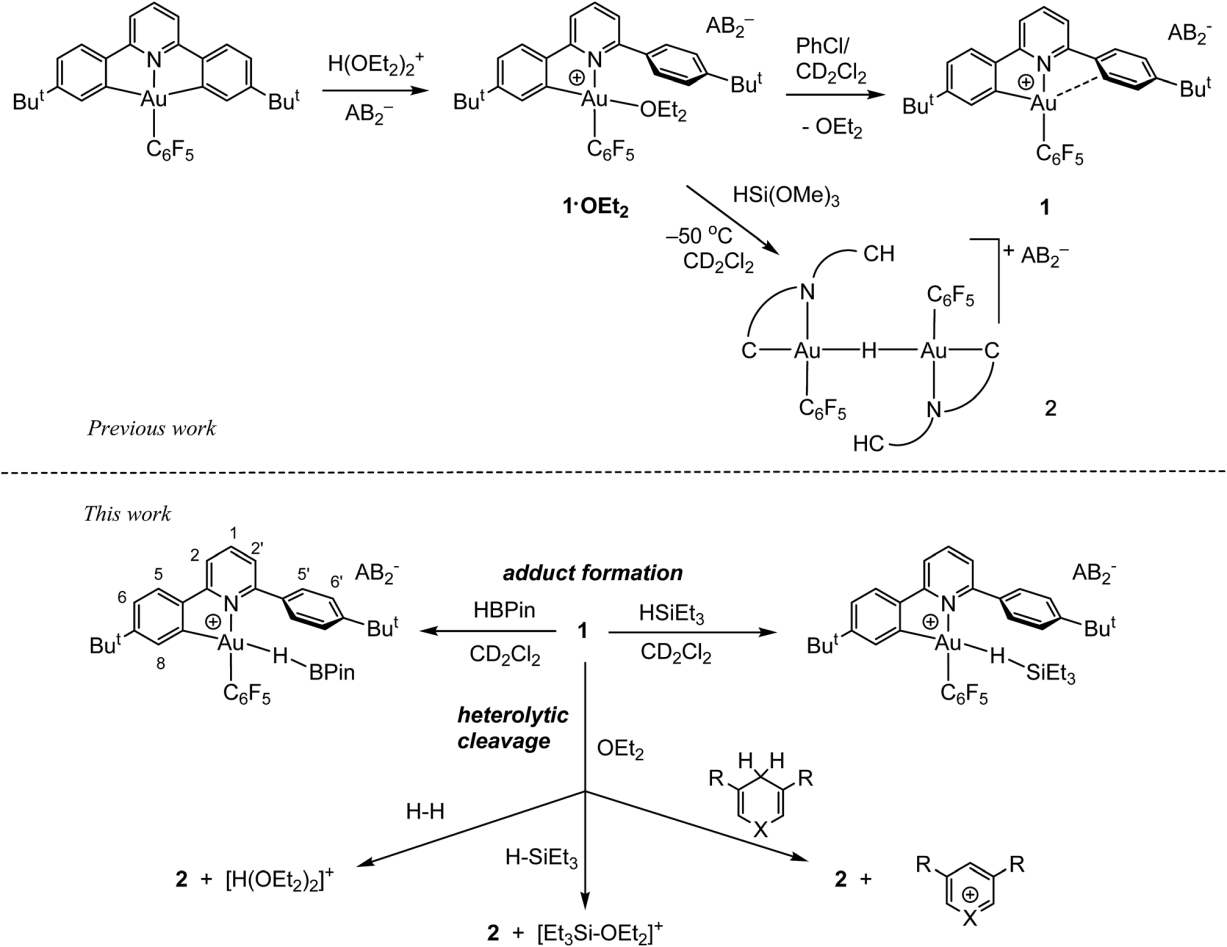
Reaction outlines including the atom labelling scheme used in NMR assignments.

As we showed recently,^42^ the reaction of the pincer complex (C^N^C)AuX with the strong Brønsted acid [H(OEt_2_)_2_]^+^[H_2_N{B(C_6_F_5_)_3_}_2_]^–^ (HAB_2_)^43^ creates the ether-stabilized C^N chelate complex [(C^N–CH)AuX(OEt_2_)]^+^AB_2_^–^ (**1·OEt_2_**) (C^N^C = 2,6-(C_6_H_3_Bu^*t*^)_2_pyridine dianion). In the present work the pentafluorophenyl derivative (X = C_6_F_5_) was chosen, firstly because this ligand provides additional stability, and secondly because the fluorine atoms can act as additional reporter nuclei and aid spectroscopic characterization. The ether ligand is labile and can be removed to afford ether-free **1** with a “dangling” –C_6_H_4_Bu^*t*^ substituent, of a “Pacman”-type structure and which is capable of supporting ligand binding to the coordination pocket, such that adducts of weak ligands with **1** are accessible which would not be feasible in C^N complexes without this dangling aryl moiety. We have also shown^44^ that treatment of **1·OEt_2_** with HSi(OMe)_3_ leads quantitatively to hydride transfer and the formation of the hydrido-bridged binuclear gold(iii) complex **2** (Scheme 1).

By contrast, we now find that gold hydride formation is preceded by formation of observable adducts between Au(iii) and H–B and H–Si bonds.

### Borane σ-complexes

In contrast to the reaction of **1·OEt_2_** with HSi(OMe)_3_, no reaction took place when this gold complex was mixed with 2 molar equiv. of HBPin at –70 °C, suggesting that the borane is unable to displace the coordinated ether ligand. However, when the solution was warmed to –20 °C, the starting material reacted slowly over a period of 30 min, affording the bridging hydride **2** quantitatively.

**Fig. 1:**
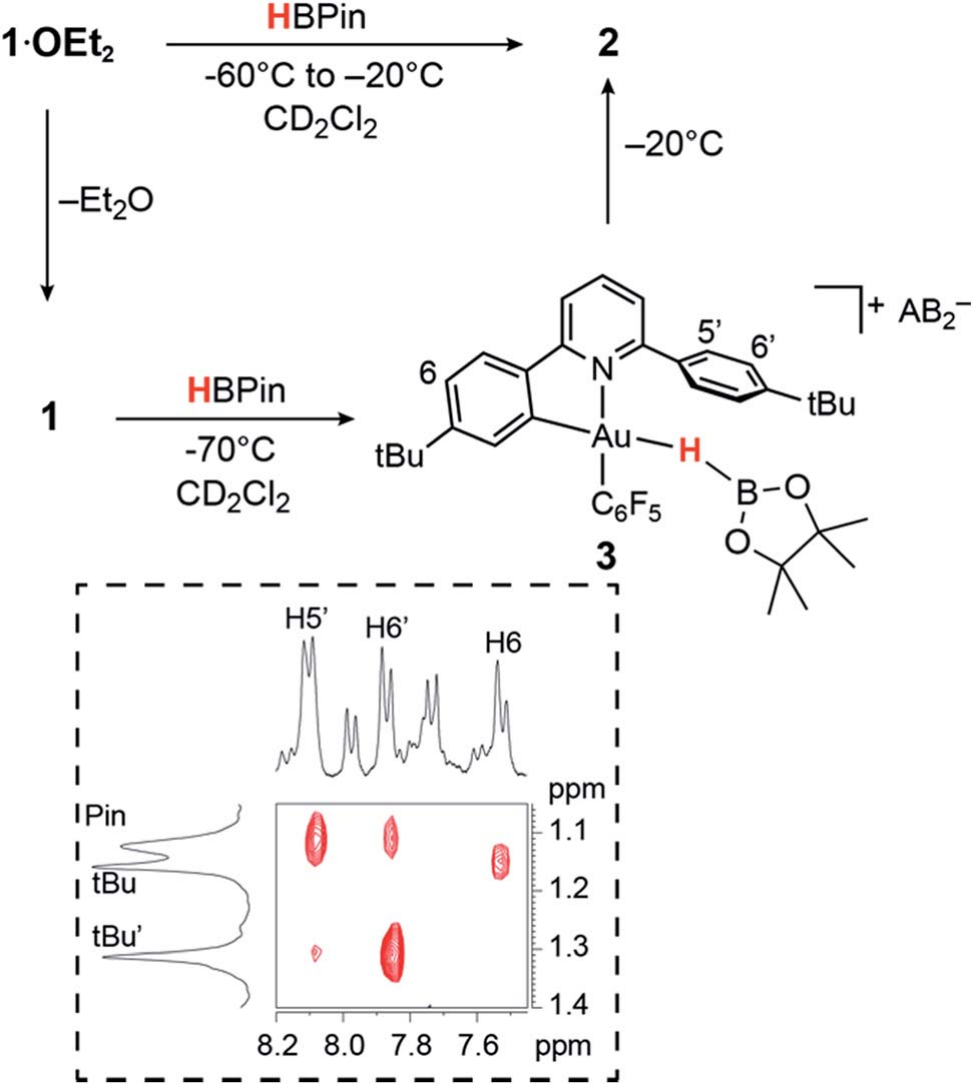
Reactivity of **1** and **1·OEt_2_** with HBPin. The inset shows a section of the ^1^H NOESY NMR spectrum of **3** (–70 °C, CD_2_Cl_2_).

On the other hand, adding 1 equiv. of HBPin to the ether-free complex **1** in dichloromethane at –70 °C immediately afforded a mixture of the hydride **2** (15%), together with a new species **3** (85%). The relative positions of the borane and the C^N ligand framework are most clearly indicated by nuclear Overhauser effect (NOE) experiments, which show selective interactions between the “dangling” –C_6_H_4_Bu^*t*^ substituent of the C^N ligand and the methyl groups of the boron-pinacolato moiety (Fig. 1). These data suggest that **3** is a gold(iii) σ-borane adduct, to our knowledge the first such observation. By contrast, as a reviewer pointed out, M–HB interactions in copper(i) and silver(i) complexes of hydroborates and anionic borane clusters are well known,^45^–^49^ while intramolecular Au(i)–HB interactions were observed in heterometallic Au–Rh carborane clusters.^50^ Further characterization of **3** is hampered by its poor thermal stability, even at very low temperatures. On warming the sample to –20 °C **3** is quickly and quantitatively converted into the hydride **2**. This suggests that heterolytic B–H splitting occurs readily without the involvement of solvent (in contrast to silanes, see below), and it seems reasonable to assume that the B–H cleavage process is assisted by the O-donor of a second molecule of HBPin. However, the ^11^B NMR spectrum is uninformative and no clear indication about the nature of the boron side-product could be obtained. The formation of a gold–HB σ-complex, as well as its lability, are in agreement with computational results (*vide infra*).

### Gold–silane σ-complexes

**Fig. 2:**
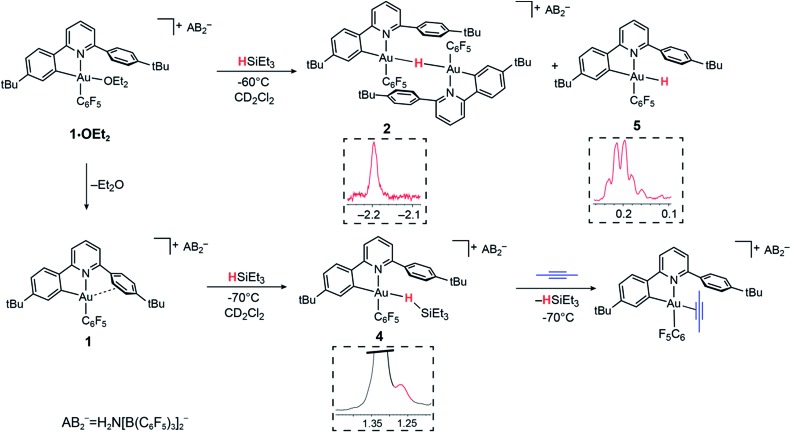
Reactivity of **1·OEt_2_** and **1** with HSiEt_3_. Insets show sections of the ^1^H NMR spectra highlighting the hydride signals.

As stated above, the reaction of **1·OEt_2_** with HSi(OMe)_3_ provided a convenient route to the μ-H gold hydride **2**. However, a very different course of reaction was observed when the ether-free cation **1** was treated with HSiEt_3_ at –70 °C in CD_2_Cl_2_. Under these conditions, there is no HSiEt_3_ cleavage and no hydride transfer. Instead, the Au(iii) σ-silane complex **4** is formed in essentially quantitative yield (Fig. 2). The identity of **4** was confirmed by multinuclear and multidimensional NMR spectroscopy. The H–Si moiety shifted by 2.19 ppm with respect to free silane, to *δ*_H_ = 1.27 ppm, while the ethyl substituents showed only moderate shifts of Δ*δ* = 0.25 and 0.28 ppm for the CH_2_ and CH_3_ groups, respectively. The coordination of HSiEt_3_ in **4** is readily reversible; resonances are broad even at –70 °C, and the ^1^H NOESY NMR spectrum shows extensive chemical exchange between free and coordinated silane. On the basis of these data it is therefore not possible to discriminate between side-on and end-on coordination modes, although DFT calculations indicate that end-on Au–H–Si bonding is preferred (*vide infra*).

The coordination of HSiEt_3_ to the Au(iii) centre in **4** is weak enough to be displaced by other weakly coordinated ligands such as 2-butyne. Under these conditions, no hydrosilylation of the alkyne was observed. Instead, the alkyne complex evolved by carbocationic alkyne cyclodimerization, as we observed previously in reactions of Au(iii) centres with sterically undemanding alkynes,^53^ while HSiEt_3_ remains unreacted.

In the presence of diethyl ether hydride transfer to gold involving heterolytic splitting of the H–SiEt_3_ bond takes place immediately, as shown by the quantitative formation of the Et_2_O-stabilized triethylsilylium cation [Et_3_SiOEt_2_]^+^ which is stable at –60 °C and was characterized spectroscopically. Relative to free Et_2_O the ethyl groups of Et_2_O in [Et_3_SiOEt_2_]^+^ are high-frequency shifted, to *δ*_H_ = 4.55 ppm (CH_2_, Δ*δ* = 1.14) and *δ*_H_ = 1.57 ppm (Δ*δ* = 0.45), respectively.

Contrary to what we observed for HSi(OMe_3_), when **1·OEt_2_** was treated with 8 equiv. of HSiEt_3_ at –30 °C, a new major product **5** was formed, accompanied by only small traces of **2** (<5%). Compound **5** is characterized by an Au–H signal at *δ*_H_ = 0.20 ppm; this chemical shift is in good agreement with the value expected for a neutral Au(iii) hydride, with H in *trans*-position to an anionic aryl-C.^44^ The ^1^H NMR signal of the hydride moiety in **5** appears as a pseudo-quartet, due to the simultaneous coupling of the hydride with one *o*-fluorine atom of the pentafluorophenyl ligand and the α proton H8 of the cyclometalated C^N chelate (for the numbering system used for NMR assignments see Scheme 1). Probing the hydrogen–fluorine heterocoupling by means of ^1^H{^19^F} and ^19^F{^1^H} experiments revealed a coupling constant ^4^*J*_HF_ = 5.7 Hz, which might arise from a through-space correlation mechanism.^54^ This pattern is not observed in **2**, where the steric constraints imposed by μ-H dimer formation hold the pentafluorophenyl rings tightly in positions perpendicular to the square-planar coordination plane. On the basis of these data **5** is identified as a neutral mononuclear gold(iii) hydride complex.

The ^19^F NMR spectrum of the mixture shows only one signal for both the *ortho* fluorine atoms of **5**, suggesting that the monomeric environment makes aryl tilting and C_6_F_5_ rotation possible, thus enabling close H–F proximity. Homocoupling between the hydride signal and H8 has previously been observed in biphenyl-based Au(iii) dihydrides Li[(C^C)AuH_2_];^44^ however, the coupling in **5** is larger (^4^*J*_HH_ = 5.0 *versus* 2.3 Hz). This suggests that minor alteration of the ligand structure has a dramatic impact on ligand flexibility and hence the spectroscopic features of this class of compounds.

Species **2** and **5** do not show chemical exchange in the ^1^H NOESY NMR spectrum recorded at –60 °C, suggesting that they form as kinetic products upon the addition of the silane. Warming the samples at temperatures above –30 °C results in the decomposition of **5** by reductive elimination, to give C_6_F_5_H. On the other hand, **2** remains unaltered up to –10 °C, as observed previously. Evidently, terminal neutral gold(iii) hydrides are less stable and more reactive than cationic H-bridged analogues. On warming the sample at room temperature C_6_F_5_H was formed quantitatively, indicating that Ar^F^–H reductive elimination is the only decomposition pathway accessible to both **2** and **5**.

The observation of Au(iii) σ-silane complexes as detectable intermediates is unprecedented in gold chemistry. There are apparently no previous examples of silane–gold complexes,^51^ and Bourissou and co-workers pointed out recently that whereas copper(i) forms intramolecular σ-Si–H adducts, gold(i) does not.^52^ Mechanistically, their formation under base-free conditions suggests that the heterolytic Si–H bond cleavage requires the presence of a Lewis base, which is capable of reacting with and stabilizing silylium cations generated after the hydride abstraction (see DFT section for details). This process contrasts with the reactions of heterogeneous gold nanoparticle hydrosilylation catalysts, for which the evidence points to the formation of H˙ radicals and Au–silyl surface species.^26^

### Hydrogen activation

The ability of gold(iii) cations to promote the heterolytic cleavage of Si–H and B–H bonds *via* a base-assisted non-redox mechanism is reminiscent of the behaviour of frustrated Lewis pairs.^55^ We therefore envisioned that same reaction principle might enable the heterolytic splitting of molecular hydrogen. When a solution of **1·OEt_2_** in CD_2_Cl_2_ was saturated with H_2_ (1 bar) at –50 °C, no reaction was observed over a period of 1 h. However, upon warming the sample to –20 °C, a slow reaction took place and gave a mixture containing **2** (41%), C_6_F_5_H (43%) and a minor component of a second hydride complex **6** with a hydride chemical shift at *δ*_H_ = –4.47 ppm (16%). At the same time the evolution of H(OEt_2_)_2_^+^ (*δ*_H_ = 16.6 ppm) was observed. These results show that heterolytic hydrogen splitting had occurred, and that some of the gold(iii) hydride formed had undergone reductive elimination of C_6_F_5_H.

**Scheme 2:**
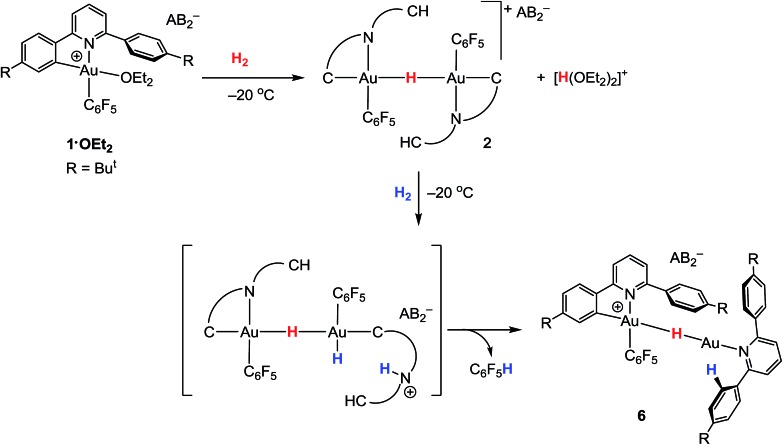
Formation of gold hydrides **2** and **6** by two successive heterolytic H–H cleavage steps.

The ^1^H NMR spectrum of the mixture shows the presence of two AA′XX′ systems for **6** at *δ*_H_ = 7.52/7.35 and *δ*_H_ = 7.18/6.66 ppm, integrating as 8 and 4 protons, respectively, which give dipole interactions with the Au–H signal in the ^1^H NOESY NMR spectrum (see ESI†). The chemical shift of the Au–H moiety in **6** matches that of Au(iii) bridging hydrides, where the hydrogen is simultaneously *trans* to an Au–C(aryl) and an Au–N(pyridine) bond.^44^ Consistent with this, the ^19^F NMR spectrum shows only one signal for the two *ortho* fluorine atoms, suggesting that the Au–C_6_F_5_ ring in **6** can rotate freely. These observations suggest that **6** is an H-bridged mixed-valence Au(iii)/Au(i) complex, formed from **2** by a second heterolytic H–H cleavage step followed by reductive elimination (Scheme 2).

**Fig. 3:**
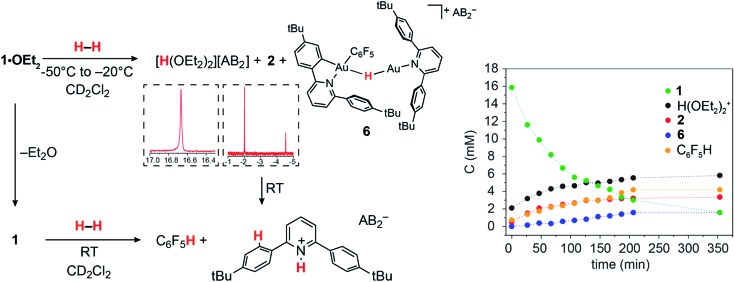
Left: Reactions of **1·OEt_2_** and of **1** with H_2_. Insets show sections of the ^1^H NMR spectrum highlighting protic and hydride signals. Right: Kinetic profile for the reaction with **1·OEt_2_** at –20 °C.

A control experiment in which pre-formed **2** was mixed with [H(OEt_2_)_2_][AB_2_] at –50 °C revealed no formation of **6**, suggesting that the latter is not the product of a side reaction of **2** with protons. We suggest that a dissociated pyridine moiety of one C^N chelate ligand in **2** acts as base and enables a second, slower heterolytic H–H cleavage step. This leads to an unstable transient Au(iii)/Au(iii) dihydride, which undergoes fast reductive elimination of C_6_F_5_H coupled with internal protodeauration of the remaining Au–aryl bond of that phenylpyridine ligand, to give **6**. Finally, upon warming the sample to room temperature a clean and quantitative conversion to C_6_F_5_H and protonated bis(4-*t*-butylphenyl)pyridine was observed, without any trace of C–C reductive elimination products (Fig. 3).^56^

Ether-free **1** is unreactive towards H_2_ at –10 °C over a period of several hours. No gold dihydrogen complex was observed, suggesting that H_2_ binding to gold(iii) is not strong enough to displace the π-interaction with the dangling aryl ring. However, **1** and H_2_ do react slowly over a period of 1 week at room temperature to generate quantitatively C_6_F_5_H and protonated bis(4-*t*-butylphenyl)pyridine, as was the case in the reaction with **1·OEt_2_**. It seems reasonable to assume that H_2_ is activated by the cooperation between gold and base (following pyridine dissociation) to give pyridinium salts and Au(iii)–H, which then decomposes upon further reaction with H_2_ as suggested in Scheme 2.

We note in this context that Corma and co-workers prepared heterogenised Schiff-base complexes of gold(iii) which proved to be highly active in olefin hydrogenation.^38^ The possible active site was probed by DFT calculations which assumed formation of Au(iii) hydrides and persistence of the Schiff-base structure. In light of the mechanistic results described above, it seems probable however that ligands with N and O-donor sites are far from innocent and may facilitate both H_2_ activation and reduction of the gold catalyst.

### Heterolytic C–H bond cleavage

**Scheme 3:**
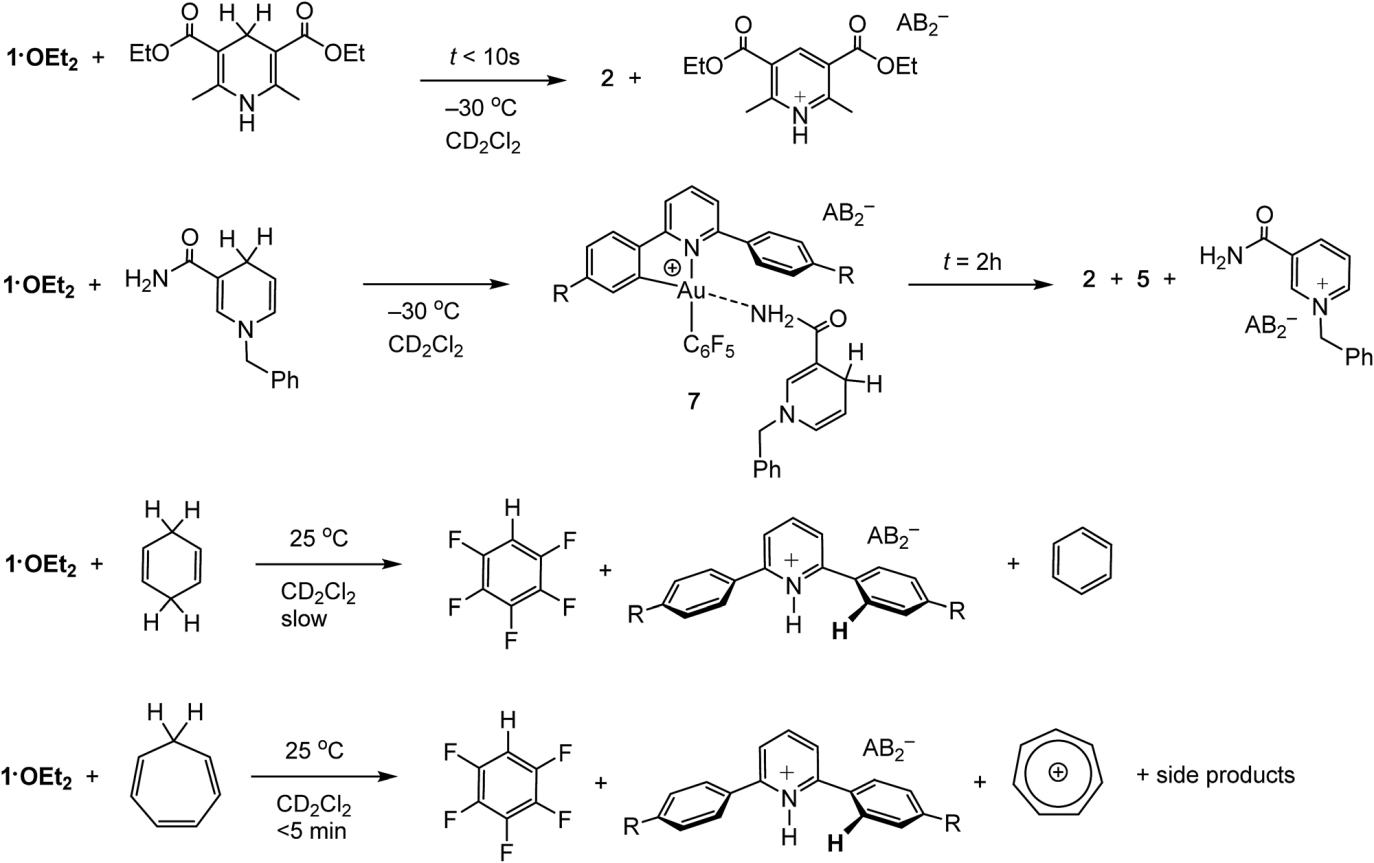
Reactions of **1·OEt_2_** with activated organic hydride donors.

Organic hydride donors can exceed dihydrogen in their ability to generate gold hydrides. For example, **1·OEt_2_** reacts with 1 equiv. of Hantzsch ester at –30 °C essentially instantaneously and much faster than with H_2_, to give **2** in quantitative yield (Scheme 3). Since the by-product is the corresponding pyridinium salt, the heterolytic C–H bond cleavage does not require the action of an external base. Indeed, ether-free **1** reacts with Hantzsch ester readily to give **2** quantitatively even at –70 °C.

*N*-Benzyl nicotinamide reacts rather more slowly under the same conditions, to give a mixture containing **2** and **5** in a 30/70 molar ratio. Interestingly, the ^1^H NMR spectrum recorded soon after mixing at –30 °C reveals the formation of the Au–nicotinamide adduct **7**. The coordination of the amide group to the metal was revealed by the chemical shift changes of the NH_2_ signal (Δ*δ* = 0.4 ppm) and those of the benzyl group and C–H moiety α to the nitrogen. The formation of **7** affects the kinetics of C–H activation and product distribution: as most of **1·OEt_2_** is captured by the nicotinamide, the concentration of gold cations with sufficient electrophilicity for hydride abstraction is reduced, leading to a slower C–H activation rate and formation of the mononuclear terminal hydride **5** as the major product.

Less activated C–H compounds react only at higher temperature and significantly more slowly. For example, 1,4-cyclohexadiene (8.5 equiv.) undergoes hydride transfer to **1·OEt_2_** over the period of 2 weeks. Since the gold hydrides formed in these reactions are not thermally stable under these conditions, their intermediacy is deduced from the appearance of the reductive elimination product, C_6_F_5_H. Under similar conditions the analogous reaction with cycloheptatriene proceeds significantly faster and is complete in <5 min, to give C_6_F_5_H and the tropylium cation C_7_H_7_^+^ in about 40% yield, accompanied by a number of side products including evidence for reductive C_6_F_5_–phenylpyridine C–C coupling (Scheme 3). NMR monitoring at –15 °C confirmed the formation of the gold hydride **2** as reaction intermediate.

The facile formation of gold hydrides from substrates such as benzyl-nicotinamide is remarkable and may have wider implications. Compounds like the Hantzsch ester and nicotinamide are well-known analogues of NADH, which is involved in electron transport in the mitochondrial respiratory chain.^57^ Many gold(iii) chelate complexes exhibit pronounced cytotoxicity, although their modes of action remain to be elucidated.^58^,^59^ The hydride abstraction ability of Au(iii) demonstrated here raises the possibility that interference in the NAD/NADH intracellular redox pathways may have to be added to the pathways responsible for the anti-tumour activity of gold(iii) compounds.

### DFT studies

The reactions of several substrates HE (E = BPin, SiMe_3_ or H) with [(C^N–CH)Au(C_6_F_5_)]^+^ (here abbreviated to LAu^+^) were studied by Density Functional Theory (DFT) calculations. Geometry optimization and thermal corrections were carried out at B3LYP/def2-SVP/PCM(CH_2_Cl_2_) level, final electronic energies from M06/cc-pVTZ/PCM(CH_2_Cl_2_), with free energies calculated at the compromise temperature of 250 K. The model ligand L has the *t*Bu substituents of the C^N–CH ligand replaced by hydrogens, and substrates were replaced by minimal models. For further details see the ESI.†

The basic reaction studied is given in eqn (1):

1 LAu^+^ + HE → [LAu···H···E]^+^ → LAuH + E^+^

Table 1 lists the most relevant free energies for different choices of E; complete listings can be found in Table S1.†

**Table 1:** Free energies of main stationary points for H–E activation by LAu^+^ (at –20 °C, in kcal mol^–1^)^*a*^

HE	LAu(HE)^+^	[LAu···H···E···B]^+^	LAuH + BE^+^	[LAu]_2_H^+^ + BE^+^
HSiMe_3_	–5.28	–0.87^*b*^	–7.15	–25.83
HBPin	–0.72	–1.71^*c*^	–4.72	–23.40
H_2_	+6.21	+9.72^*b*^	+10.51^*e*^	–16.06^*f*^
PyCarH	—	+3.67^*b*^ ^,^^*d*^	–7.57^*d*^	–26.25
CHDH	—	+16.22^*b*^ ^,^^*d*^	+15.83^*d*^	–2.85
Ph_3_CH	—	—	+19.24^*d*^	+0.56
PhCH_3_	—	^*g*^	+45.80^*d*^	+27.12

^*a*^LAu^+^ + HE as reference (0 kcal mol^–1^); B = OMe_2_; reference energy of LAu(B)^+^ is –8.86 kcal mol^–1^ if the reaction is done in presence of ether.

^*b*^Transition state.

^*c*^Intermediate (local minimum).

^*d*^No assistance by ether.

^*e*^Not separated products but complex LAuH···BE^+^.

^*f*^Forming H[OMe_2_]_2_^+^ instead of HOMe_2_^+^.

^*g*^Barrierless to reactants.

**Scheme 4:**
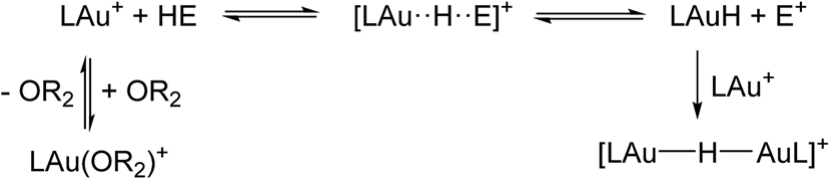
H–E activation equilibria.

Before going into specific reactions, a few general points should be noted: (i) several reactions start with the ether complex LAu(OR_2_)^+^, see Scheme 4. The initial dissociation of coordinated ether costs 8.9 kcal mol^–1^. (ii) The initially produced LAuH would have the hydride *trans* to the aryl-C atom of the C^N–CH ligand. The isomer with H *trans* to N(pyridine) is actually preferred by 6.5 kcal mol^–1^, although it is not clear how such an isomerization would happen. However, any LAuH formed can be quickly trapped by unreacted LAu^+^ to form the dimer [(LAu)_2_(μ-H)]^+^, for which a structure with H *trans* to both aryl-C atoms is preferred. (iii) In many of the cases studied the basic reaction (1) is endergonic. However, the trapping of the initial product LAuH by LAu^+^ adds –18.7 kcal mol^–1^ to the reaction free energy, pulling the equilibrium to the right (Scheme 4).

(iv) The formation of free E^+^ is energetically unfavourable. However, the presence of a Lewis base B (ether in this case) may help trap and stabilize the H abstraction product E^+^ as an adduct BE^+^. Stabilization by B may start at various stages of reaction (1): by initial coordination of B to HE, or by having B as a nucleophile displacing LAuH from E, or even after hydride displacement has already completed. This results in a set of similar but subtly different “mechanisms” as detailed below.

**Fig. 4:**
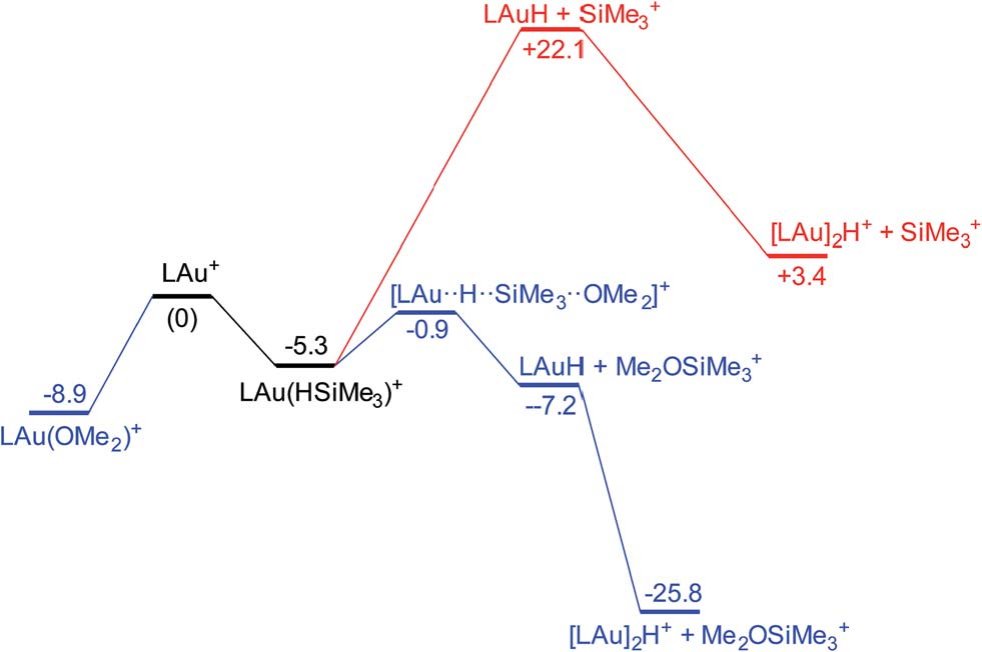
Free energy profile of the reaction on LAu^+^ with HSiMe_3_ in the presence (black and blue parts) and absence (black and red parts) of OMe_2_.

**Fig. 5:**
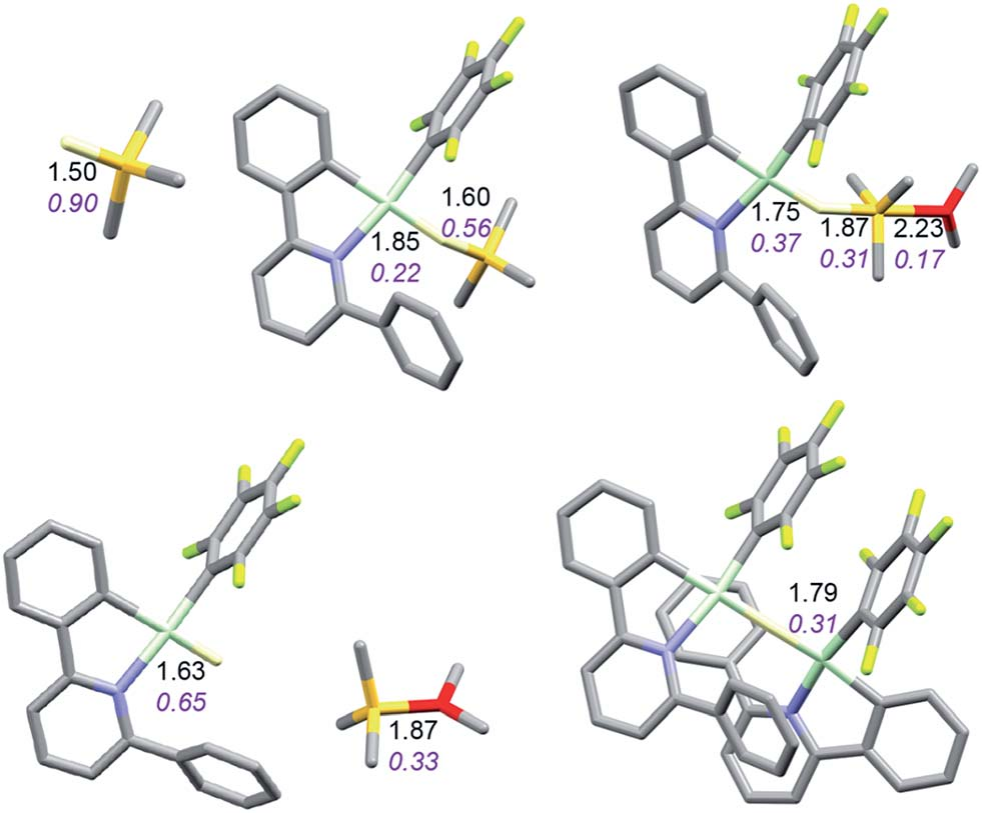
Structure of the silane complex [LAu(HSiMe_3_)]^+^ and activation of HSiMe_3_ by LAu^+^/OMe_2_. Bond lengths in Å, Wiberg bond indexes shown in purple and italics.

It is convenient to start with a silane (HSiMe_3_) substrate. The relevant energy profile is shown in Fig. 4. Reaction of HSiMe_3_ with LAu^+^ produces a weakly bound silane complex (–5.3 kcal mol^–1^ relative to “naked” LAu^+^), but this low binding energy is misleading: both Si–H and Au–H bonds are clearly elongated relative to the parent molecules HSiMe_3_ and LAuH, indicating extensive electronic reorganization to the separated hydrides. Relevant bond lengths and Wiberg bond indexes (WBIs) are shown in Fig. 5; the WBI suggests that at this stage H transfer has progressed to about 30%. Simple dissociation of SiMe_3_^+^ is not feasible, and even trapping of LAuH by LAu^+^ is not enough to make this reaction exergonic. However, if the same reaction is carried out in the presence of ether, an S_N_2-like attack of OMe_2_ on the Si atom of the coordinated silane leading to loss of [Me_2_OSiMe_3_]^+^ is remarkably easy, requiring only 4.4 kcal mol^–1^. The rate-limiting step for this process is actually the initial ether dissociation, which costs 8.9 kcal mol^–1^, corresponding to a reaction that is extremely fast even at –60 °C.

**Fig. 6:**
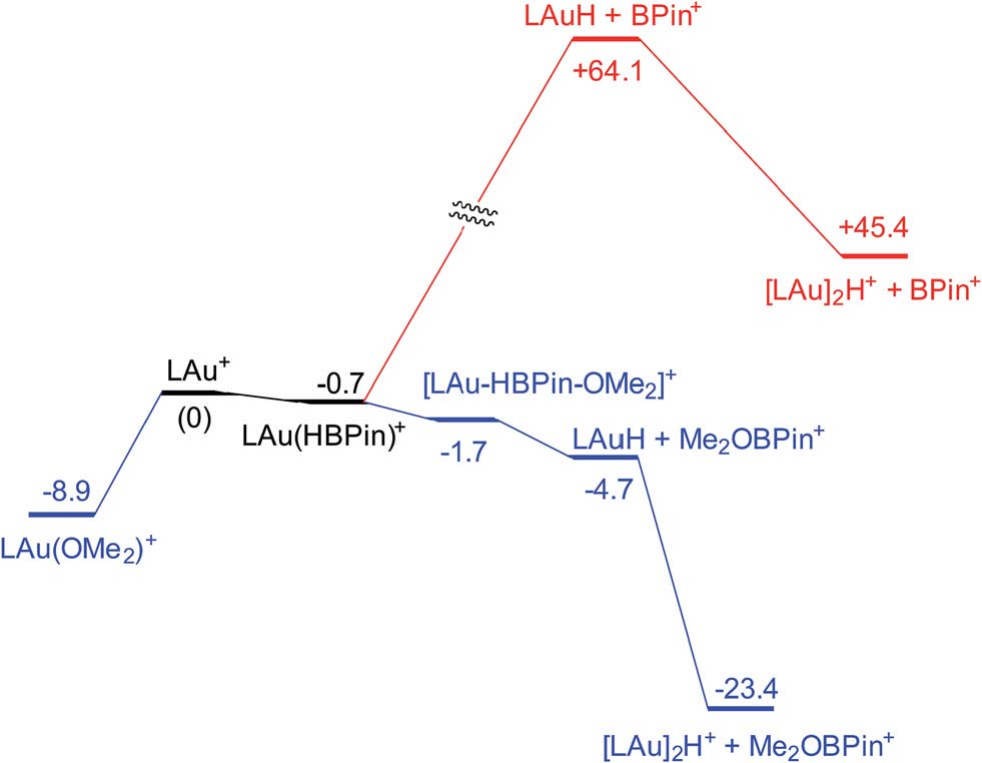
Free energy profile (kcal mol^–1^) for the reaction of HBPin with LAu^+^ in the presence (black and blue parts) and absence (black and red parts) of ether OMe_2_.

**Fig. 7:**
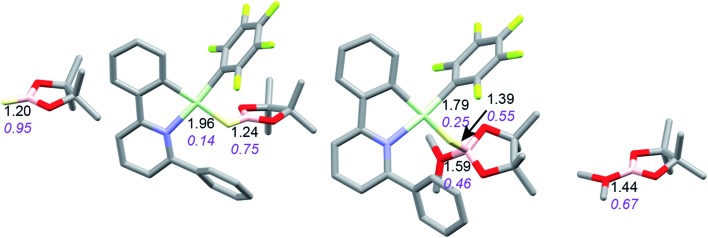
Activation of HBPin by LAu^+^/OMe_2_. Bond lengths in Å, Wiberg bond indexes shown in purple and italics.

Coordination of borane HBPin to LAu^+^ (see profile in Fig. 6) is even weaker than coordination of silane (less than 1 kcal mol^–1^). The resulting species is best regarded as a borane σ-complex, with a short B–H bond and a large Au–H distance, as is clear from the B–H/Au–H bond distances and the corresponding WBIs (Fig. 7). Again, direct dissociation of BPin^+^ is not feasible. Approach of OMe_2_ does not result in displacement of the hydride from B, the ether simply coordinates weakly (∼1 kcal mol^–1^) to the B atom. This results in considerable reorganization and has the effect of making the borane a better hydride donor, as is clear from the elongation of the B–H bond and considerable shortening of the Au–H bond: the ether complex can now be seen as a gold hydride donating density to the B atom of (Me_2_O)BPin^+^. Finally, dissociation of (Me_2_O)BPin^+^ produces the free gold hydride, to be captured by LAu^+^. The subtle difference with the silane case is that here the LAu(HBPin)(OMe_2_)^+^ represents a local minimum (intermediate) instead of an S_N_2-like transition state. Similar to the silane case, this is predicted to be a very fast reaction.

**Fig. 8:**
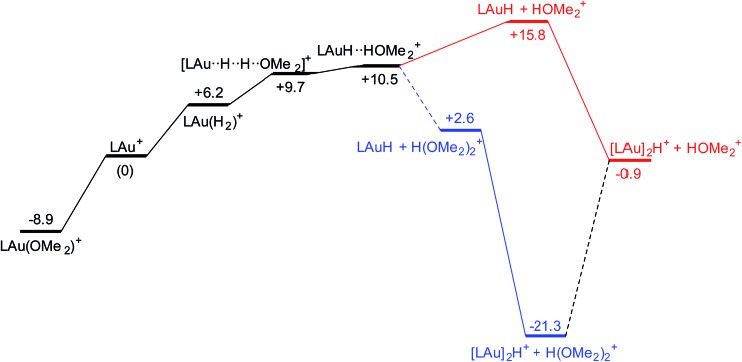
Free energy profile (kcal mol^–1^) for the reaction of H_2_ with LAu^+^ assisted by one (black and red parts) and two (black and blue parts) molecules of ether OMe_2_.

**Fig. 9:**
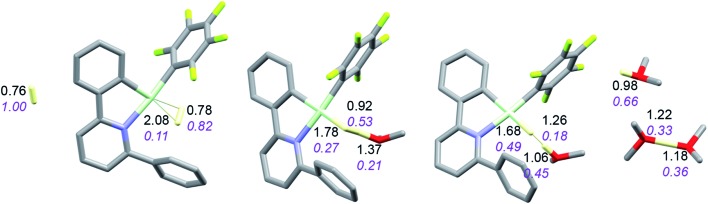
Activation of H_2_ by LAu^+^/OMe_2_. Bond lengths in Å, Wiberg bond indexes shown in purple and italics.

The reaction with dihydrogen obviously requires a base (here OMe_2_) to bind the proton released in the heterolytic cleavage of H_2_. However, one molecule of ether is apparently not enough. The free energy profile (Fig. 8) shows that H_2_ binding is endergonic by 6.1 kcal mol^–1^. The coordinated dihydrogen molecule is hardly activated, as is clear from bond lengths and WBIs in Fig. 9. From there, attack by OMe_2_ at one of the hydrogen atoms of H_2_ leads to heterolytic cleavage, forming the tightly bound pair [LAuH·HOMe_2_]^+^ at +10.5 kcal mol^–1^ relative to ether-free LAu^+^. Dissociation into separate LAuH and HOMe_2_^+^ would cost another 5.3 kcal mol^–1^, leading to an effective barrier of 24.7 kcal mol^–1^ which would correspond to a reaction that is slow at room temperature. More likely, the tightly bound pair [LAuH·HOMe_2_]^+^ binds another molecule of ether, forming a less tightly bound combination of LAuH and [H(OMe_2_)_2_]^+^,^42^ after which separation and trapping of LAuH would lead to the final products. This variation would have an effective barrier of 19.4 kcal mol^–1^ which would be more compatible with a reaction that is slow at –20 °C. The main difference with the silane and borane variations is that for H_2_ coordination of the substrate is endergonic and hence contributes to the effective reaction barrier.

The reaction with dihydropyridine 3,5-dicarboxylate (PyCarH) does not require further assistance by a base: after initial dissociation of ether from the starting complex LAu(OMe_2_)^+^, the actual H abstraction step has a barrier of 3.4 kcal mol^–1^. This leads to an effective activation free energy of 12.3 kcal mol^–1^, indicating a rather fast reaction. On the other hand, hydride abstraction from toluene or triphenylmethane is thermodynamically not feasible, while abstraction from 1,4-cyclohexadiene (CHDH) in the absence of ether is just on the edge; this reaction is exergonic by only 2.9 kcal mol^–1^ if LAuH trapping is included, with a calculated barrier of 16.2 kcal mol^–1^. This is in agreement with the observed slow reaction at room temperature.

To summarize, LAu^+^ is a strong hydride acceptor, made even more potent by trapping of the initial product LAuH to form the binuclear H-bridged complex [(LAu)_2_(μ-H)]^+^. It is comparable in strength to the 3,5-dicarboxylatopyridinium cation, and considerably weaker than the cyclohexadienyl and trityl cations. A common feature of the reactions studied is the surprisingly low barrier for the H transfer step, typically less than 5 kcal mol^–1^ relative to the substrate complex. These results illustrate how effective gold(iii) is in polarizing the H–E bond in preparation for heterolytic cleavage.

## Conclusion

Gold(iii) complexes have been shown to be able to form σ-complexes with boranes and silanes, species that have long been postulated as the first steps in gold catalysed hydroboration and hydrosilylation reactions. These complexes are sufficiently stable for spectroscopic characterisation. DFT calculations suggest end-on Au–H–E bonding, with binding free energies of the order of 1–5 kcal mol^–1^. The key to these weak adducts is the use of the gold(iii) chelate complex [(C^N–CH)Au(C_6_F_5_)]^+^, which contains a gold–arene π-interaction to the “dangling” aryl substituent which is weak enough to be displaced by H–E, while at the same time the coordinated substrate is protected by this “Pacman”-type ligand structure. In the presence of ether as base heterolytic H–E bond scission is facile. The same holds for the activation of dihydrogen. The observations reported here on gold(iii) mediated H_2_ activation follow the same principle as postulated in earlier computational studies on hydrogen activation by gold(i) compounds,^39^ where a polar protic solvent like ethanol was shown to be required in the activation process. These results suggest that for gold catalysts in which the active centre is positively polarized to some extent, heterolytic rather than homolytic H–E and H–H bond cleavage processes prevail, at least in the presence of weakly basic solvents or reagents. The range of H-donors for gold could be extended to activated hydrocarbons, where in favourable cases C–H bond activation was shown to be orders of magnitude faster than dihydrogen activation. Overall, these reactions have provided routes to unprecedented types of gold complexes and serve to illustrate the remarkable capacity of gold to polarize and activate substrate H–E bonds.

## Conflicts of interest

There are no conflicts to declare.

## Supplementary Material

Supplementary information

## References

[cit1] Hashmi A. S. K., Hutchings G. J. (2006). Angew. Chem., Int. Ed..

[cit2] Schmidbaur H., Schier A. (2012). Arabian J. Sci. Eng..

[cit3] Raubenheimer H. G., Schmidbaur H. (2011). S. Afr. J. Sci..

[cit4] Fürstner A., Davies P. W. (2007). Angew. Chem., Int. Ed..

[cit5] Leyva-Pérez A., Corma A. (2012). Angew. Chem., Int. Ed..

[cit6] Hopkinson M. N., Tlahuext-Aca A., Glorius F. (2016). Acc. Chem. Res..

[cit7] Miró J., del Pozo C. (2016). Chem. Rev..

[cit8] Joost M., Amgoune A., Bourissou D. (2015). Angew. Chem., Int. Ed..

[cit9] Roşca D.-A., Wright J. A., Bochmann M. (2015). Dalton Trans..

[cit10] Blons C., Amgoune A., Bourissou D. (2018). Dalton Trans..

[cit11] Claus P. (2005). Appl. Catal., A.

[cit12] McEwan L., Julius M., Roberts S., Fletcher J. C. Q. (2010). Gold Bull..

[cit13] Pan M., Brush A. J., Pozun Z. D., Chul Ham H., Yu W.-Y., Henkelman G., Hwang G. S., Mullins C. B. (2013). Chem. Soc. Rev..

[cit14] Pan M., Gong J., Dong G., Mullins C. B. (2014). Acc. Chem. Res..

[cit15] Cardenas-Lizana F., Keane M. A. (2013). J. Mater. Sci..

[cit16] Keane M. A., Li M., Collado L., Cárdenas-Lizana F. (2018). React. Kinet., Mech. Catal..

[cit17] Corma A., Serna P. (2006). Science.

[cit18] Corma A., Serna P., Garcia H. (2007). J. Am. Chem. Soc..

[cit19] Serna P., Boronat M., Corma A. (2011). Top. Catal..

[cit20] Vilhanova B., van Bokhoven J. A., Ranocchiari M. (2017). Adv. Synth. Catal..

[cit21] Malta G., Kondrat S. A., Freakley S. J., Davies C. J., Lu L., Dawson S., Thetford A., Gibson E. K., Morgan D. J., Jones W., Wells P. P., Johnston P., Catlow C. R. A., Kiely C. J., Hutchings G. J. (2017). Science.

[cit22] Yang M., Li S., Wang Y., Herron J. A., Xu Y., Allard L. F., Lee S., Huang J., Mavrikakis M., Flytzani-Stephanopoulos M. (2014). Science.

[cit23] Corma A., Gonzalez-Arellano C., Iglesias M., Sanchez F. (2007). Angew. Chem., Int. Ed..

[cit24] Saridakis I., Kidonakis M., Stratakis M. (2018). ChemCatChem.

[cit25] Kidonakis M., Stratakis M. (2015). Org. Lett..

[cit26] Li H., Guo H., Li Z., Wu C., Li J., Zhao C., Guo S., Ding Y., He W., Li Y. (2018). Chem. Sci..

[cit27] Chen Z., Zhang Q., Chen W., Dong J., Yao H., Zhang X., Tong X., Wang D., Peng Q., Chen C., He W., Li Y. (2018). Adv. Mater..

[cit28] Bhattacharjee R., Datta A. (2017). J. Phys. Chem. C.

[cit29] Lv H., Zhan J.-H., Cai Y.-B., Yu Y., Wang B., Zhang J.-L. (2012). J. Am. Chem. Soc..

[cit30] Debono N., Iglesias M., Sanchez F. (2007). Adv. Synth. Catal..

[cit31] Satoh Y., Igarashi M., Sato K., Shimada S. (2017). ACS Catal..

[cit32] Leyva A., Zhang X., Corma A. (2009). Chem. Commun..

[cit33] Silverwood I. P., Rogers S. M., Callear S. K., Parker S. F., Catlow C. R. A. (2016). Chem. Commun..

[cit34] Manzoli M., Chiorino A., Vindigni F., Boccuzzi F. (2012). Catal. Today.

[cit35] Bus E., Miller J. T., van Bokhoven J. A. (2005). J. Phys. Chem. B.

[cit36] Corma A., Boronat M., González S., Illas F. (2007). Chem. Commun..

[cit37] Takale B. S., Feng X., Lu Y., Bao M., Jin T., Minato T., Yamamoto Y. (2016). J. Am. Chem. Soc..

[cit38] Comas-Vives A., González-Arellano C., Corma A., Iglesias M., Sánchez F., Ujaque G. (2006). J. Am. Chem. Soc..

[cit39] Comas-Vives A., Ujaque G. (2013). J. Am. Chem. Soc..

[cit40] Fiorio J. L., López N., Rossi L. M. (2017). ACS Catal..

[cit41] Fiorio J. L., Gonçalves R. V., Teixeira-Neto E., Ortuño M. A., López N., Rossi L. M. (2018). ACS Catal..

[cit42] Rocchigiani L., Fernandez-Cestau J., Budzelaar P. H. M., Bochmann M. (2017). Chem. Commun..

[cit43] Lancaster S. J., Rodriguez A., Lara-Sanchez A., Hannant M. D., Walker D. A., Hughes D. L., Bochmann M. (2002). Organometallics.

[cit44] Rocchigiani L., Fernandez-Cestau J., Chambrier I., Hrobarik P., Bochmann M. (2018). J. Am. Chem. Soc..

[cit45] Dobrott R. D., Lipscomb W. N. (1962). J. Chem. Phys..

[cit46] Hertz R. K., Goetze S., Shore S. G. (1979). Inorg. Chem..

[cit47] Bommer J. C., Morse K. W. (1980). Inorg. Chem..

[cit48] Beckett M. A., Jones P. W. (1997). Synth. React. Inorg. Met.-Org. Chem..

[cit49] Nuss G., Saischek G., Harum B. N., Volpe M., Belaj F., Mösch-Zanetti N. C. (2011). Inorg. Chem..

[cit50] Carr N., Gimeno M. C., Goldberg J. E., Pilotti M. U., Stone F. G. A., Topalğalu I. (1990). J. Chem. Soc., Dalton Trans..

[cit51] Corey J. Y. (2011). Chem. Rev..

[cit52] Joost M., Mallet-Ladeira S., Miqueu K., Amgoune A., Bourissou D. (2013). Organometallics.

[cit53] Rocchigiani L., Fernandez-Cestau J., Agonigi G., Chambrier I., Budzelaar P. H. M., Bochmann M. (2017). Angew. Chem., Int. Ed..

[cit54] Hierso J.-C. (2014). Chem. Rev..

[cit55] Stephan D. W., Erker G. (2015). Angew. Chem., Int. Ed..

[cit56] Rocchigiani L., Fernandez-Cestau J., Budzelaar P. H. M., Bochmann M. (2018). Chem.–Eur. J..

[cit57] Ohnishi T. Ohnishi S. T. Salernoa J. C., Biol. Chem., 2018, 399 , 1249 –1264 , , and cited references . 30243012 10.1515/hsz-2018-0164

[cit58] Bertrand B. Williams M. R. M. Bochmann M., Chem.–Eur. J., 2018, 24 , 11840 –11851 , , and cited references . 29575433 10.1002/chem.201800981

[cit59] Zou T. T., Lum C. T., Lok C. N., Zhang J. J., Che C. M. (2015). Chem. Soc. Rev..

