# Supplementary material for: Heterolytic bond activation at gold: evidence for gold(iii) H–B, H–Si complexes, H–H and H–C cleavage
Source: Chem Sci. 2019 Jan 16;10(9):2633–42. doi: 10.1039/c8sc05229h (PMC6425858; doi:10.1039/c8sc05229h)
Supplement: Supplementary file 1 [file SC-010-C8SC05229H-s001.pdf]

Electronic Supplementary Material (ESI) for Chemical Science.
This journal is © The Royal Society of Chemistry 2019

Heterolytic Bond Activation at Gold: Evidence for Gold(III) H-B, H-Si Complexes, H-H and H-C Cleavage

Luca Rocchigiani,[a*] Peter H. M. Budzelaar,[b*] Manfred Bochmann[a*]

Supporting Information

1. Experimental.

When required, manipulations were performed using standard Schlenk techniques under dry argon or using a nitrogen-filled MBraun Unilab glovebox equipped with a high capacity recirculator (<1.0 ppm O_2 and H_2O). Argon was purified by passing through columns of supported P_2O_5 with moisture indicator and of activated 4 Å molecular sieves. Anhydrous solvents were freshly distilled from the appropriate drying agents and degassed. Triethylsilane (99%), pinacolborane (97%), 1,4-cyclohexadiene (97%), diethyl 1,4-dihydro-2,6-dimethyl-3,5-pyridinedicarboxylate (95%), 1-benzyl-1,4-dihydronicotinamide (97%) and cycloheptatriene (95%) were obtained by Sigma Aldrich and dried, when necessary. CD_2Cl_2 (Apollo Scientific), was freeze-pump-thaw degassed over CaH_2, distilled and stored over activated 4Å molecular sieves. (C^N^C)AuC_6F_5[S1] and $[H(OEt_2)_2][H_2N\{B(C_6F_5)_3\}_2]$[S2] were synthesized according to literature procedures.

Experiments with H_2 were performed on a dedicated Schlenk line interfaced with a Parker Domnic Hunter hydrogen generator (H_2 purity >99.9995%) at 1 atmosphere.

^{1}H, $^{1}H\{^{19}F\}$ ^{1}H PGSE, ^{19}F, $^{19}F\{^{1}H\}$, $^{13}C\{^{1}H\}$, ^{1}H COSY, ^{1}H NOESY, $^{1}H,^{13}C$ HMQC, and $^{1}H,^{13}C$ HMBC NMR experiments have been recorded on a Bruker DPX–300 spectrometer equipped with a ^{1}H,BB smartprobe and Z-gradients. ^{1}H NMR spectra are referenced to the residual protons of the deuterated solvent. ^{13}C NMR spectra are referenced to the D-coupled ^{13}C signals of the solvent. ^{19}F NMR spectra are referenced to an external standard of $CFCl_3$.

References:

S1 D.-A. Roşca, D. A. Smith and M. Bochmann, *Chem. Commun.*, 2012, **48**, 7247–7249.

S2 S. J. Lancaster, A. Rodriguez, A. Lara-Sanchez, M. D. Hannant, D. A. Walker, D. L. Hughes and M. Bochmann, *Organometallics* 2002, **21**, 451 – 453

2. Reactions with HBPin

Reaction with HBPin in the presence of Et_2O:

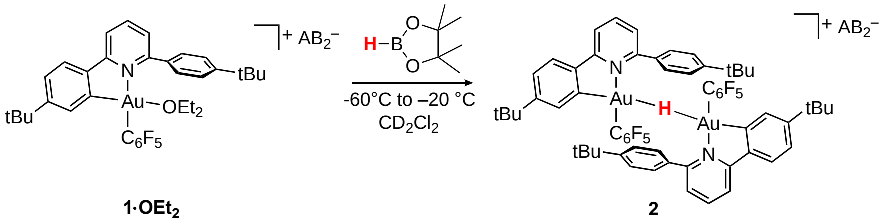

1·OEt₂ 2

1·OEt$_2$ was generated at room temperature in the glovebox by reacting 5 mg of (C^N^C)AuC_6F_5 and 1 equivalent of [H(OEt_2)$_2$][H_2N(B(C_6F_5)$_3$)$_2$] within a screw cap NMR tube in CD_2Cl_2. The NMR tube was then inserted into a cold bath at –78°C and a solution of HBPin (3 equivalents in CD_2Cl_2) was injected through the septum of the NMR tube by a micrometric syringe. The solution was quickly shaken and inserted into the pre-cooled NMR probe and analyzed at -60°C. Quantitative conversion of **1·OEt$_2$** into **2** was observed upon warming the sample up to –20°C for 30 minutes.

Reaction with HBPin under base-free conditions:

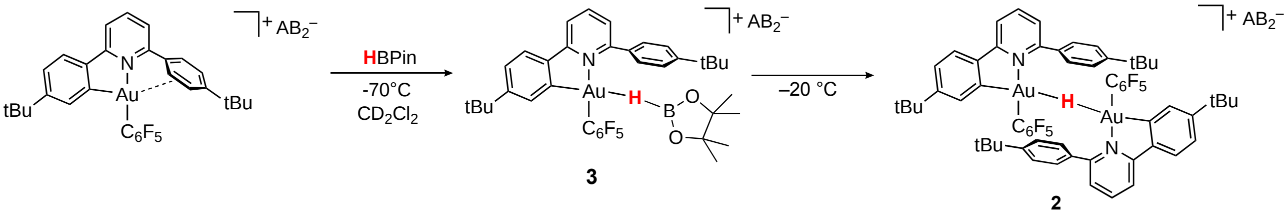

3 2

1·OEt$_2$ was generated at room temperature in the glovebox by reacting 7.5 mg of (C^N^C)AuC_6F_5 and 1 equivalent of [H(OEt_2)$_2$][H_2N(B(C_6F_5)$_3$)$_2$] within a J-Young NMR tube in C_6D_5Cl. The tube was then dried under vacuum to remove any trace of Et_2O and redissolved in CD_2Cl_2. The resultant solution was transferred into a screw-cap NMR tube and inserted in a cold bath at -78°C. A solution containing 1 molar equivalents of HBPin was injected through the septum and the sample was quickly shaken before inserting the tube in the precooled NMR probe at -70°C. The first ^{1}H NMR spectrum revealed the formation of a mixture of **3** (80%) and **2** (20%). Data for **3**: ^{1}H NMR (300.13 MHz, CD_2Cl_2, 203 K, *J* values in Hz): 8.35 (t, $^3J_{HH}$=8.1, 1H, H1), 8.06 (br d, 3H, H2+H5'), 7.93 (d, $^3J_{HH}$=8.1, 1H, H2'), 7.70 (d, partially overlapped with **2**, H5), 7.48 (d, partially overlapped with **2**, H6), 6.49 (s, 1H, H8), 5.62 (br s, NH_2), 1.27 (s, 9H, CMe_3), 1.12 (s, 9H, CMe_3'), 1.08 ppm (s, 12H, 10). ^{19}F NMR (275.55 MHz, CD_2Cl_2, 203 K, *J* values in Hz): –119.1 (br s, 2F, *o*–F C_6F_5), –130.9 (br s, *o*–F [H_2N{B(C_6F_5)$_3$}]), –136.4 (br s, *o*–F [H_2N{B(C_6F_5)$_3$}]), –150.9 (t, $^3J_{FF}$=21.0, 1F, *p*–F C_6F_5), –159.1 (br t *p*–F [H_2N{B(C_6F_5)$_3$}]), –157.9 (m, 2F, *m*–F C_6F_5), –164.7 ppm (br s, *m*–F [H_2N{B(C_6F_5)$_3$}]).

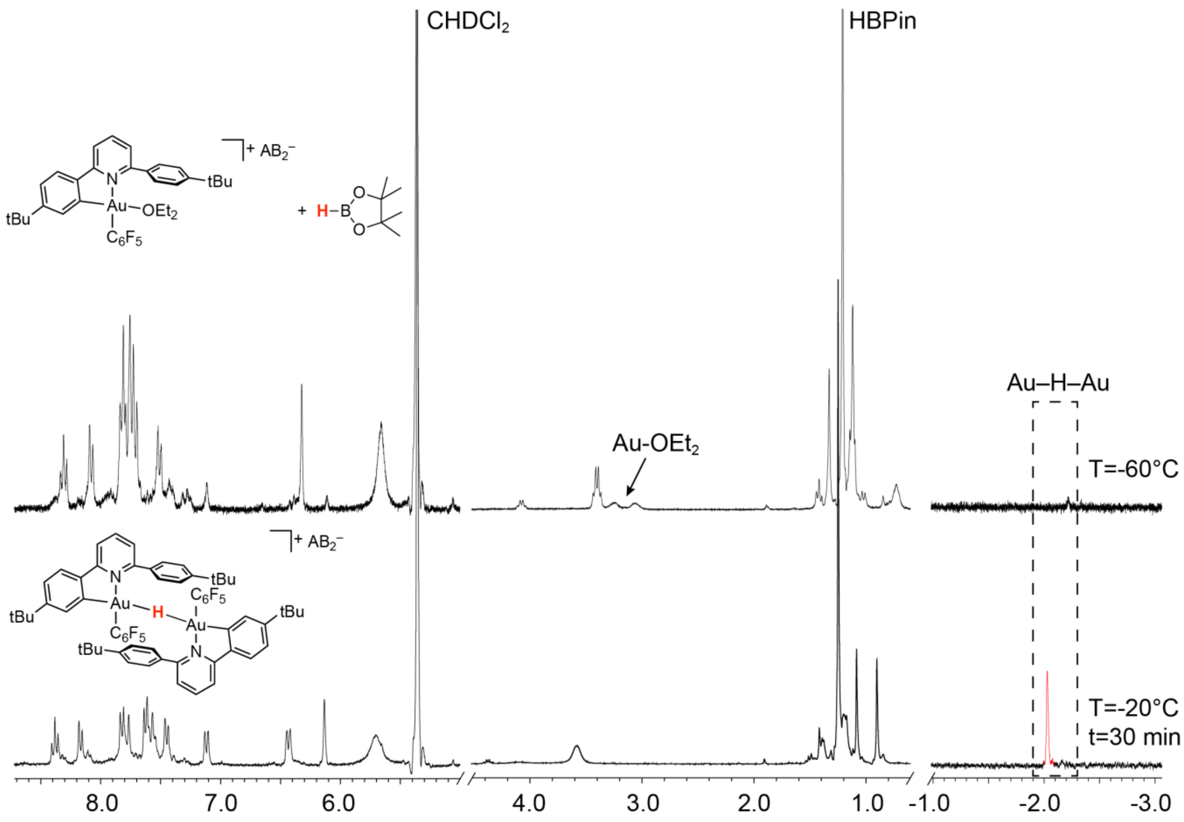

Figure S1. Evolution of the ^{1}H NMR spectrum of **1·OEt₂** (CD_2Cl_2, 213K) upon the addition of 3 equivalents of HBPin and warming up to –20 °C.

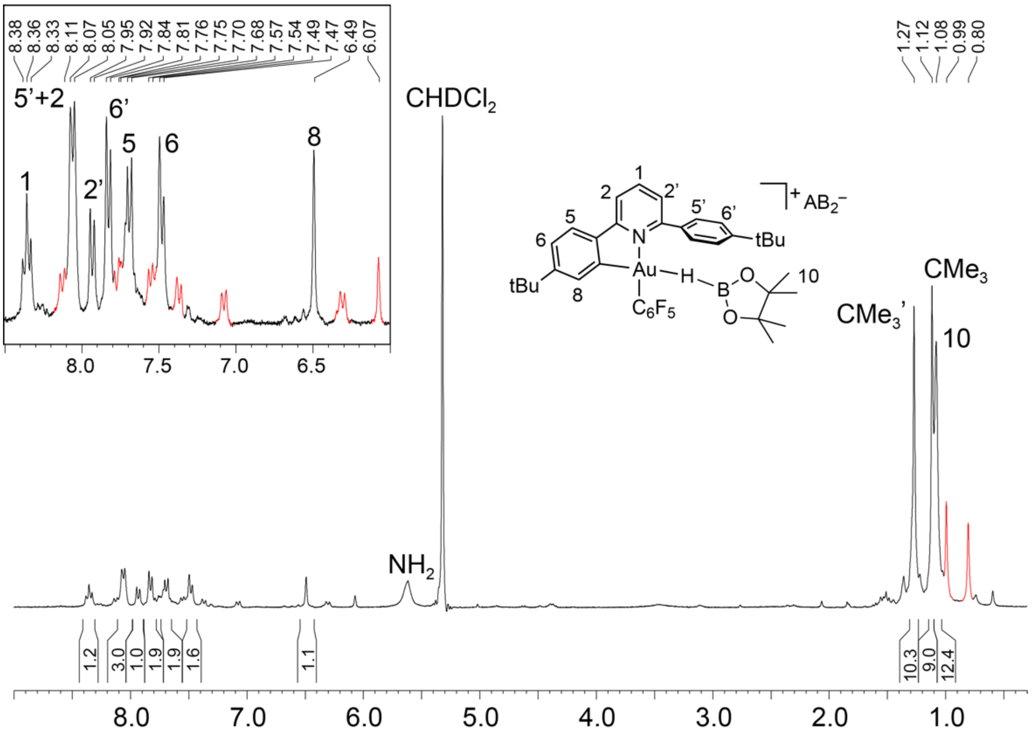

Figure S2. ^{1}H NMR spectrum of **3** (CD_2Cl_2, 203K); red trace denotes traces of **2**.

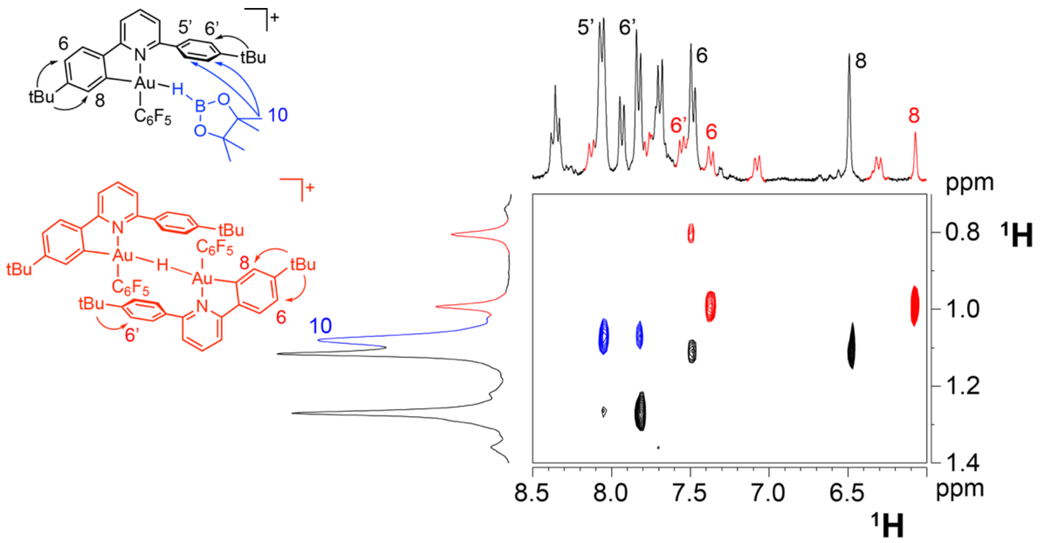

Figure S3. A section of the ^{1}H NOESY NMR spectrum of **5** (CD_2Cl_2, 203K); red trace denotes traces of **2**.

3. Reactions with $HSiEt_3$

Reaction with $HSiEt_3$ in the presence of Et_2O:

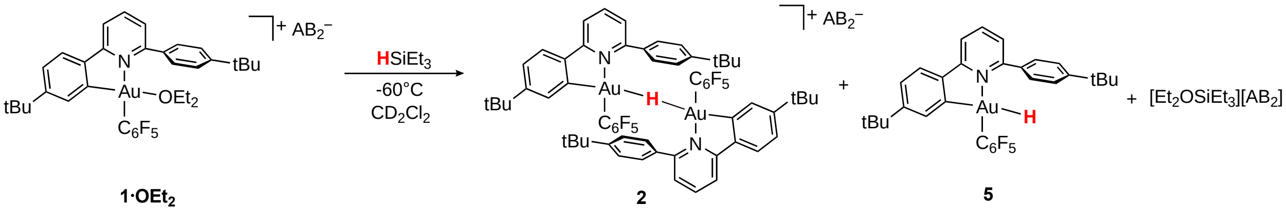

1·OEt₂ **2** **5**

1·OEt$_2$ was generated at room temperature in the glovebox by reacting 5 mg of (C^N^C)AuC_6F_5 and 1 equivalent of $[H(OEt_2)_2][H_2N(B(C_6F_5)_3)_2]$ within a screw cap NMR tube in CD_2Cl_2. The NMR tube was then inserted into a cold bath at –78°C and a solution of $HSiEt_3$ in CD_2Cl_2 was injected through the septum of the NMR tube by a micrometric syringe. The solution was quickly shaken and inserted into the pre-cooled NMR probe and analyzed. The experiment has been performed under different experimental conditions: when 8 molar equivalents of $HSiEt_3$ are used at –30°C, **2** and **5** were obtained in a molar **2**/**5** ratio >95/5; when the amount of silane was decreased to 2 equivalents and the temperature lowered to -60°C, the **2**/**5** ratio amounted to 85/15. Due to the poor signal to noise ratio, ^{13}C NMR data for **5** are obtained indirectly through HMBC and HMQC experiments. Data for **3**: ^{1}H NMR (300.13 MHz, CD_2Cl_2, 213 K, J values in Hz): δ 8.13 (t, $^{3}J_{HH}$=7.9, 1H, H1), 8.00 (d, $^{3}J_{HH}$=7.9, 1H, H1), 7.79 (d, partially overlapped with **2**, H5), 7.61 (d, partially overlapped with **2**, H2’), 7.52 (AB system, 4H, H5’+H6’), 7.35 (dd, $^{3}J_{HH}$=8.1, $^{4}J_{HH}$=2.0, 1H, H6), 6.96 (dd, $^{4}J_{HH}$=5.0, $^{4}J_{HH}$=1.7, 1H, H8), 1.33 (s, 9H, ^{t}Bu’), 1.16 (s, 9H, ^{t}Bu), 0.20 ppm (ps q, $^{4}J_{HH}$=5.0, $^{4}J_{HF}$=5.7, 1H, Au–H). ^{13}C{^{1}H} NMR (300.13 MHz, CD_2Cl_2, 213 K): 170.5 (s, C9), 167.1 (s, C3), 162.5 (s, C3’), 154.1 (s, C7), 152.3 (s, C7’), 145.0 (s, C4), 141.6 (s, C1), 141.5 (s, C4’), 130.6 (s, C8), 129.3 (s, C5’ or C6’), 125.8 (s, C5), 125.1 (s, C6’ or C5’), 124.3 (s, C6), 124.0 (s, C2’), 119.3 (s, C2), 35.0 (s, CMe_3 + CMe_3’), 31.3 (s, CMe_3’), 30.7 ppm (s, CMe_3). ^{19}F NMR (275.55 MHz, CD_2Cl_2, 213 K, J values in Hz): –120.2 (m, 2F, *o*–F C_6F_5), –159.7 (t, $^{3}J_{FF}$=20.7, 1F, *p*–F C_6F_5), –162.7 ppm (m, 2F, *m*–F C_6F_5).

Reaction with $HSiEt_3$ under base-free conditions:

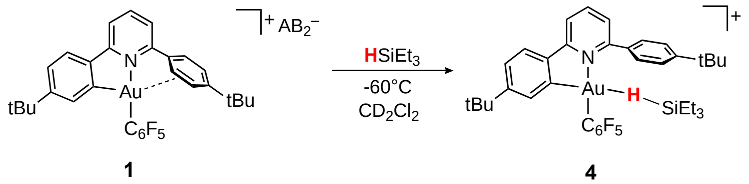

1·OEt₂ was generated at room temperature in the glovebox by reacting 7.5 mg of (C^N^C)AuC_6F_5 and 1 equivalent of $[H(OEt_2)_2][H_2N(B(C_6F_5)_3)_2]$ within a J-Young NMR tube in C_6D_5Cl. The tube was then dried under vacuum to remove any trace of Et_2O and the residue was dissolved in CD_2Cl_2. The resultant solution was transferred into a screw-cap NMR tube and inserted into a cold bath at -78 °C. A solution containing 2 molar equivalents of $HSiEt_3$ was injected through the septum. The sample was quickly shaken before inserting the tube into the precooled NMR probe at -60 °C.

Data for **4**: ^{1}H NMR (300.13 MHz, CD_2Cl_2, 203 K, *J* values in Hz): 8.33 (t, $^3J_{HH}$=8.1, 1H, H1), 8.07 (d, $^3J_{HH}$=8.1, 1H, H1), 7.90 (br m, 3H, H5'+H2'), 7.74 (br m, 3H, H6'+H5), 7.49 (br d, 1H, H6), 6.63 (s, 1H, H8), 5.62 (br s, 2H, NH_2), 1.32 (s, 9H, CMe_3'), 1.26 (br s, 1H, Au–*H*–Si), 1.13 (s, 9H, CMe_3), 0.85 (m, partially overlapped with $HSiEt_3$, H11), 0.85 ppm (br m, 6H, H10). ^{13}C{^{1}H} NMR (300.13 MHz, CD_2Cl_2, 203 K): 161.1 (s, C3), 159.2 (s, C3'), 156.8 (s, C7), 156.2 (s, C7'), 150.6 (s, C9), 147.4 (br d, $^1J_{CF}$=234.5, *o*–C $[H_2N\{B(C_6F_5)_3\}]$), 144.7 (s, C1), 139.5 (s, C4), 138.8 (br d, $^1J_{CF}$=245.5, *p*–C $[H_2N\{B(C_6F_5)_3\}]$), 136.4 (br d, $^1J_{CF}$=245.5, *p*–C $[H_2N\{B(C_6F_5)_3\}]$), 134.5 (s, C4'), 130.6 (s, C8), 129.7 (s, C5'), 127.8 (s, C6'), 127.4 (s, C6), 126.2 (s, C2'), 120.2 (s, C2), 35.7 (s, *C*Me_3), 35.4 (s, *C*Me_3'), 30.8 (s, C*Me_3*'), 30.5 (s, C*Me_3*), 6.96 (s, C11), 2.0 ppm (s, C10).

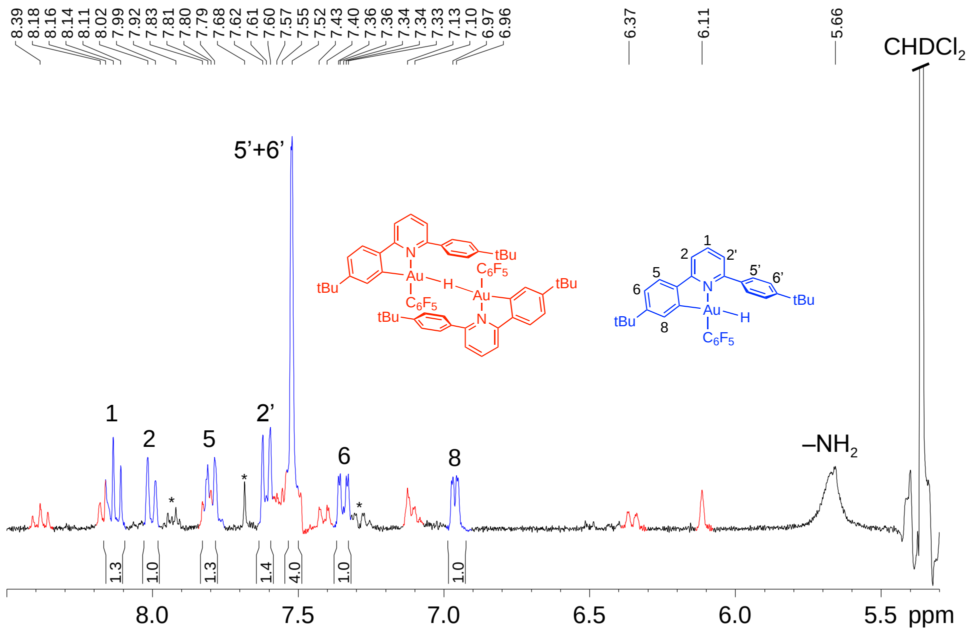

Figure S4. A section of the ^{1}H NMR spectrum obtained after mixing **1·OEt₂** and $HSiEt_3$ (CD_2Cl_2, 213K); * denote traces of reduction products.

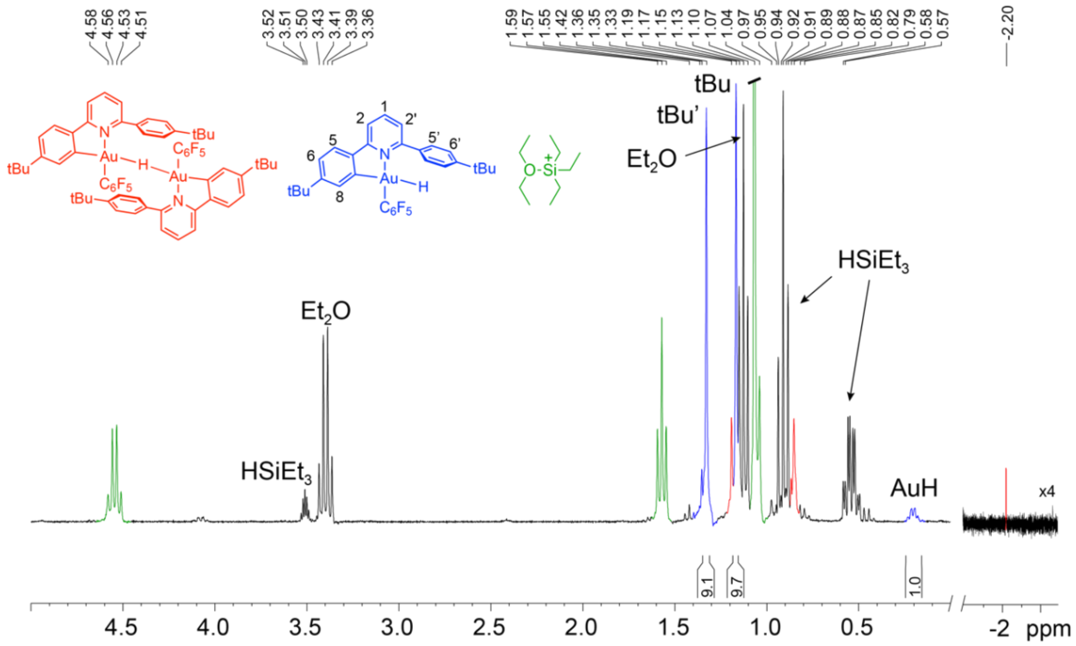

Figure S5. A section of the ^{1}H NMR spectrum obtained after mixing **1·OEt$_2$** and $HSiEt_3$ (CD_2Cl_2, 213K); * denote traces of reduction products.

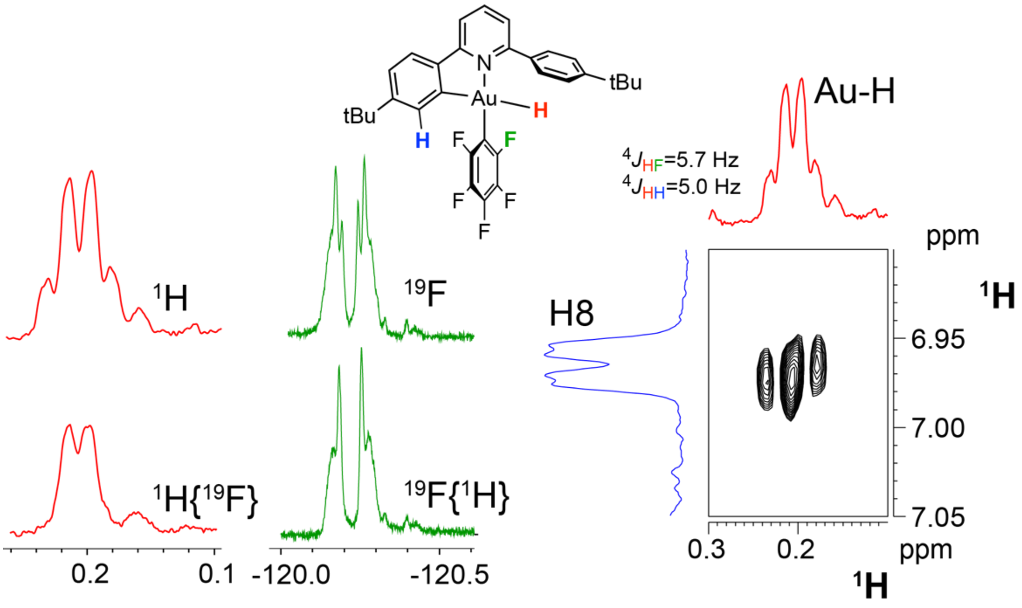

Figure S6. Sections of the ^{1}H, ^{1}H{^{19}F}, ^{19}F, ^{19}F{^{1}H} and ^{1}H COSY NMR spectra obtained after mixing **1** and $HSiEt_3$ (CD_2Cl_2, 213K) showing the scalar coupling pattern of the hydride signal in complex **5**.

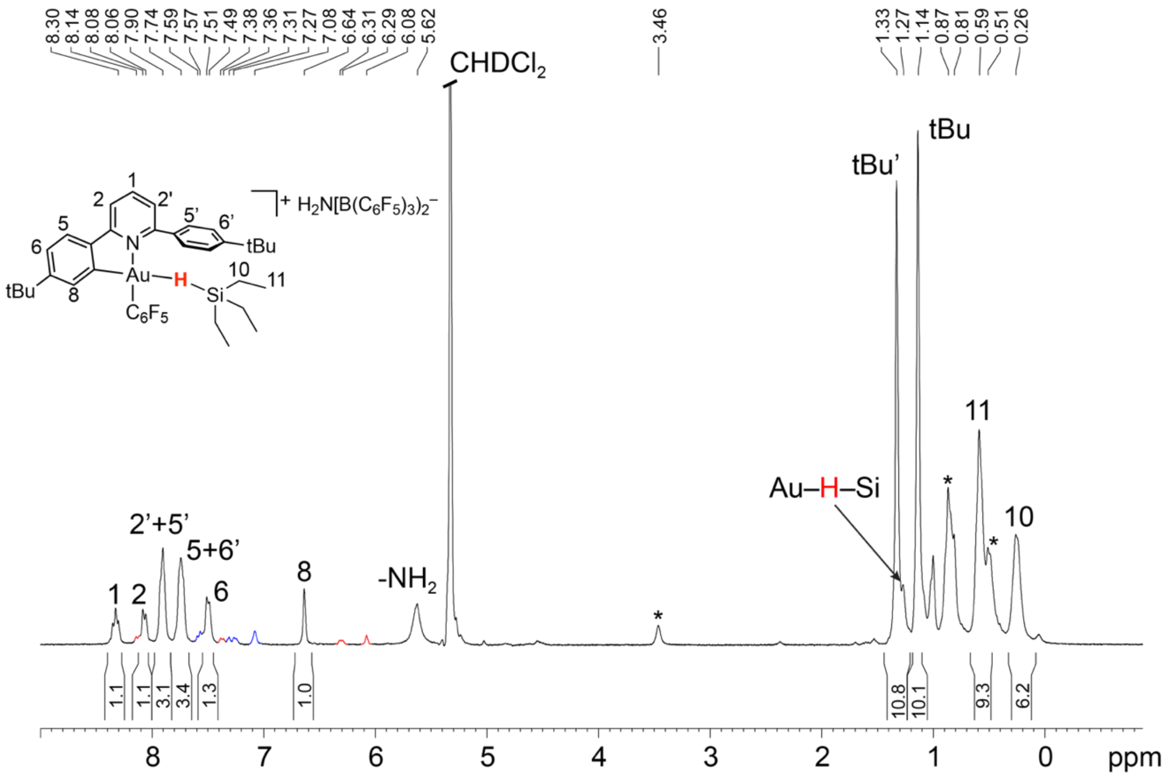

Figure S7. ^{1}H NMR spectrum obtained after mixing **1** and $HSiEt_3$ (CD_2Cl_2, 203K); red signals correspond to traces of hydride **2**, blue signals are relative to unreacted (C^N^C)AuC_6F_5. * denotes free $HSiEt_3$.

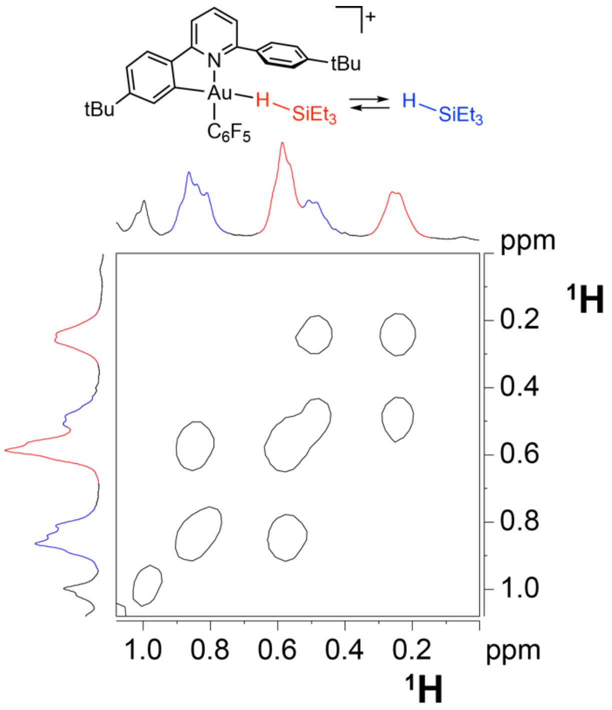

Figure S8. A section of the ^{1}H NOESY NMR spectrum obtained after mixing **1** and $HSiEt_3$ (CD_2Cl_2, 203K) showing the presence of chemical exchange between free and bound $HSiEt_3$.

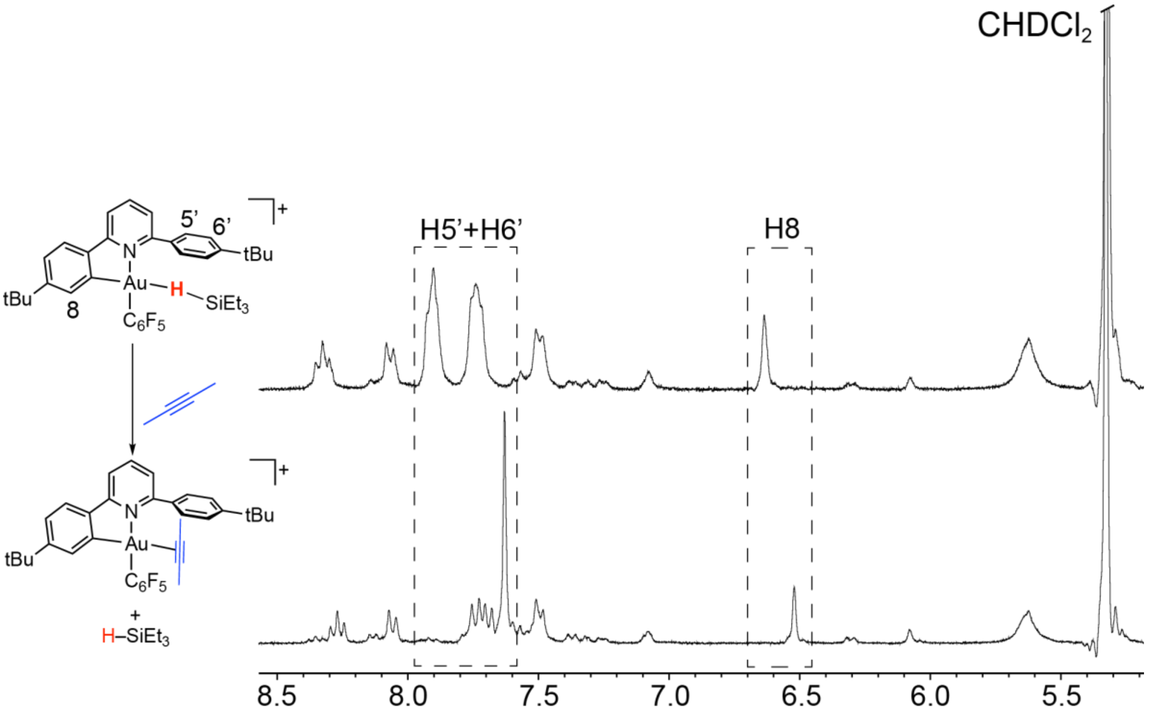

Figure S9. Evolution of the ^{1}H NMR spectrum of **4** (CD_2Cl_2, 203K) upon the addition of 3 equivalents of 2-butyne. The boxes highlight spectral change for the 5', 6' and H8 regions.

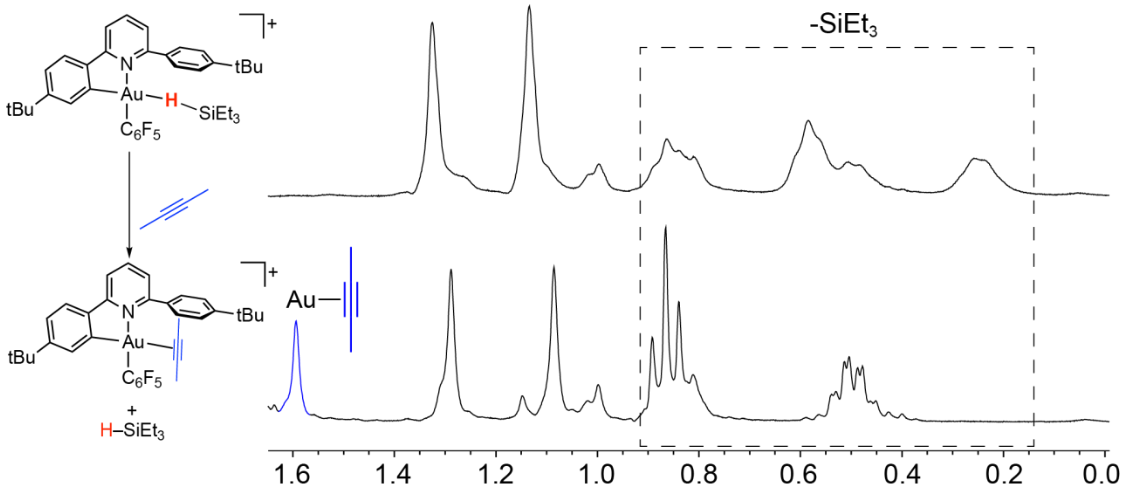

Figure S10. Evolution of the ^{1}H NMR spectrum of **4** (CD_2Cl_2, 203K) upon the addition of 3 equivalents of 2-butyne.

4. Reactions with H_2

Reaction with H_2 in the presence of Et_2O:

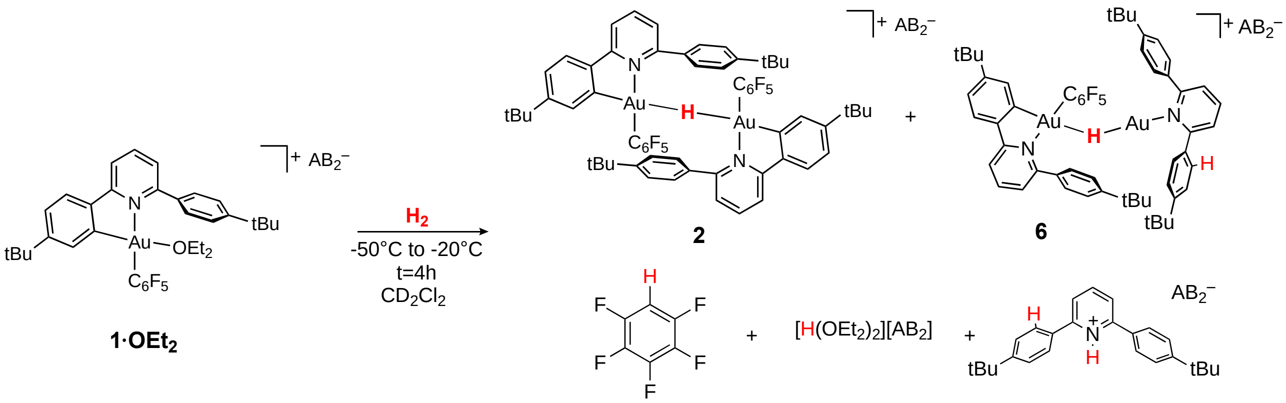

1·OEt$_2$ was generated at room temperature in the glovebox by reacting 7.5 mg of (C^N^C)AuC_6F_5 and 1 equivalent of $[H(OEt_2)_2][H_2N(B(C_6F_5)_3)_2]$ within a J-Young NMR tube in CD_2Cl_2. Successively, the sample was interfaced to the vacuum line, freeze-pump-thaw degassed 3 times and exposed to H_2 (1 atm) for 1 minute at 77 K. The frozen solution was allowed to melt at 195 K and transferred into the pre-cooled NMR probe at 223 K.

Reaction with H_2 under base-free conditions:

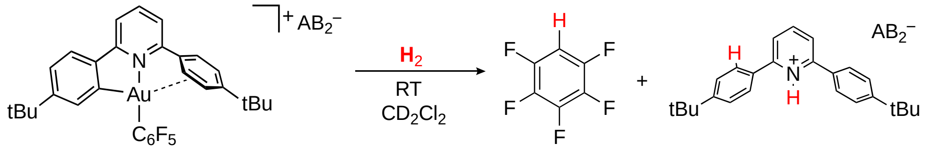

1·OEt$_2$ was generated at room temperature in the glovebox by reacting 7.5 mg of (C^N^C)AuC_6F_5 and 1 equivalent of $[H(OEt_2)_2][H_2N(B(C_6F_5)_3)_2]$ within a J-Young NMR tube in C_6D_5Cl. The tube was then dried under vacuum to remove any trace of Et_2O and redissolved in CD_2Cl_2. Successively, the sample was interfaced to the vacuum line, freeze-pump-thaw degassed 3 times and exposed to H_2 (1 atm) for 1 minute at 77 K. The frozen solution was allowed to melt at 195 K and transferred into the pre-cooled NMR probe at 223 K. No reaction was observed over the course of several hours, so the sample was warmed up to room temperature and left reacting for 1 week.

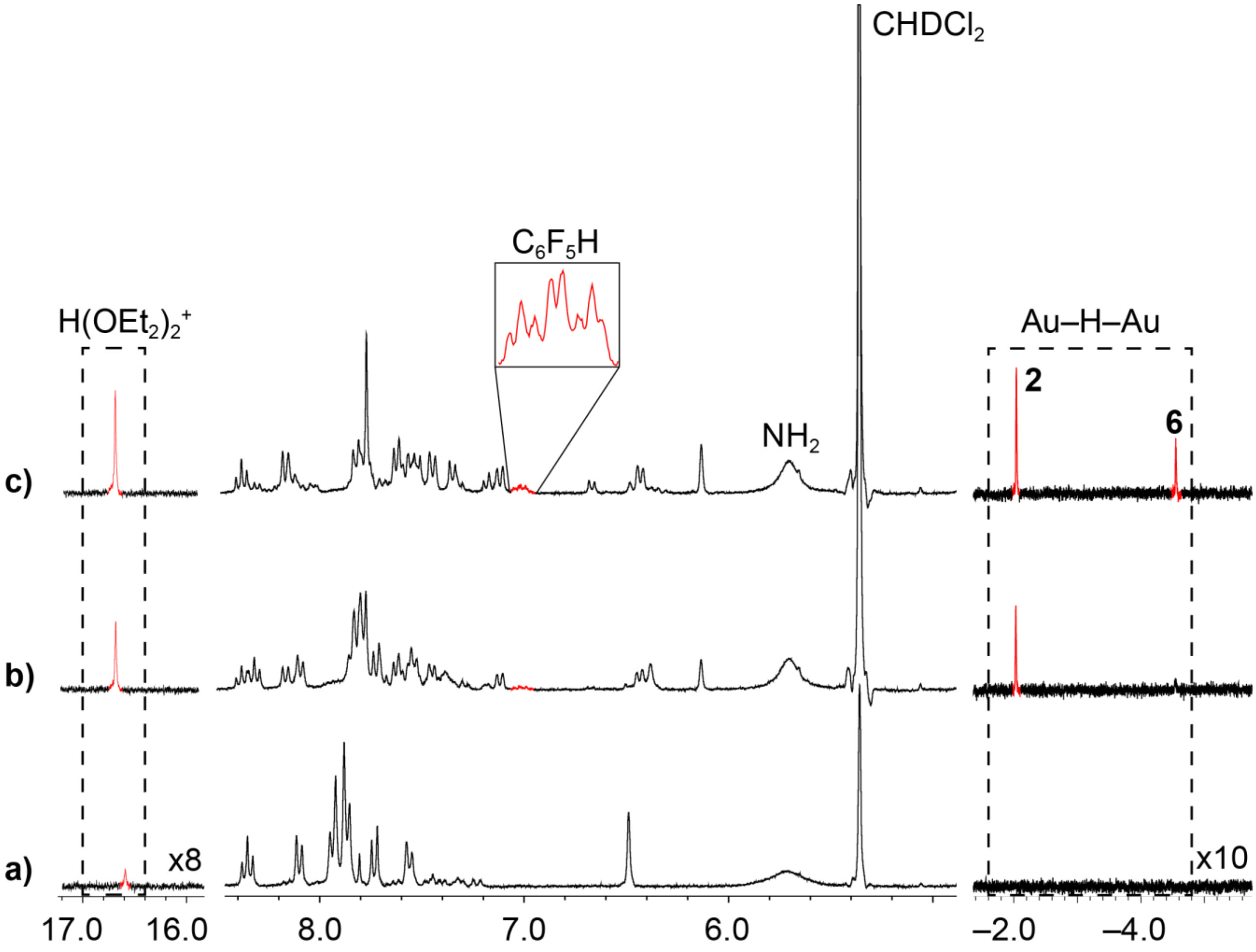

Figure S11. Evolution of three sections of the ^{1}H NMR of **1·OEt$_2$** (a) upon exposure to H_2 at –20 °C after 2 hours (b) and 4 hours (c).

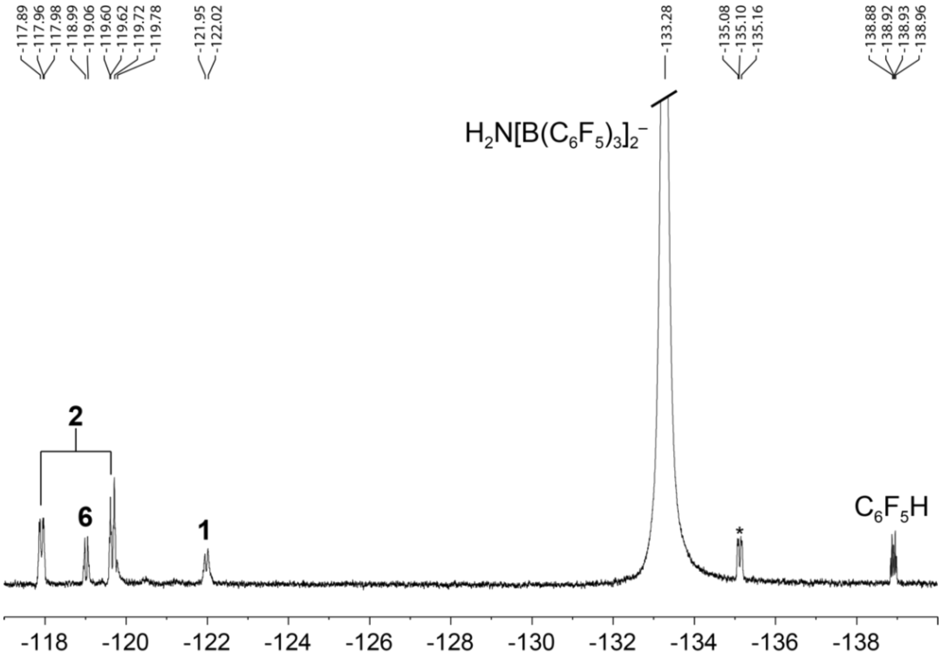

Figure S12. A section of the ^{19}F NMR spectrum obtained during the reaction of **1·OEt$_2$** with H_2 (CD_2Cl_2, 253K). Asterisk denote inert minor impurity in $H_2N[B(C_6F_5)_3]_2^-$.

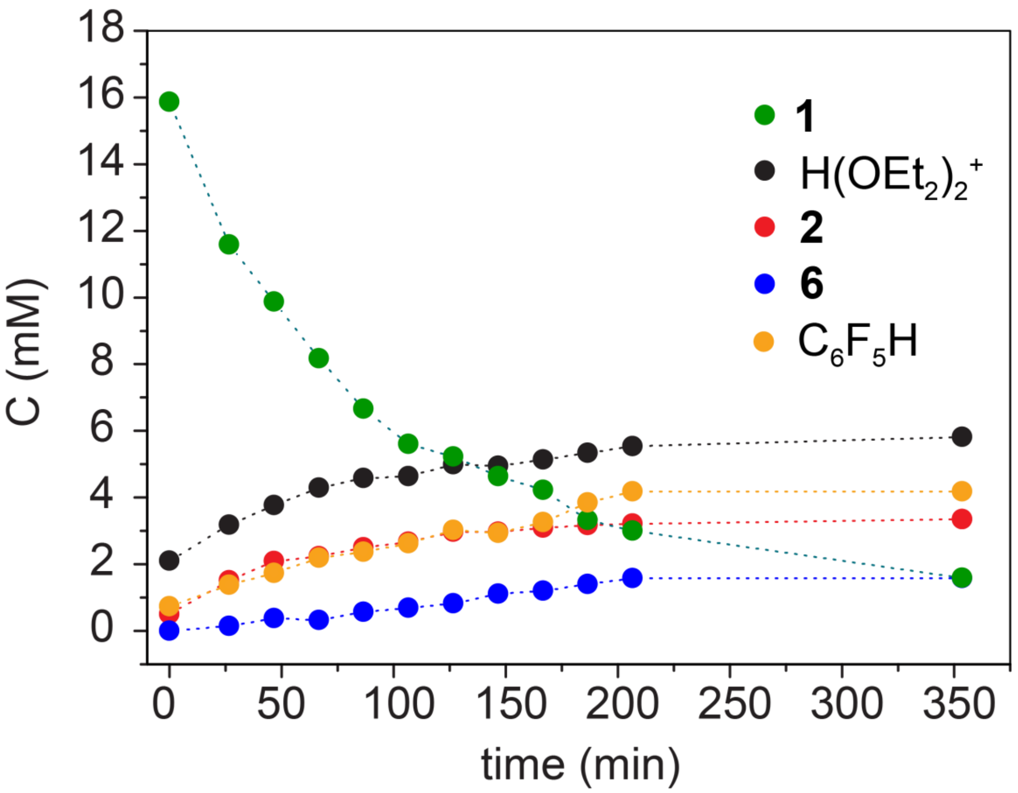

Figure S13. Kinetic profile of the reaction between **1·OEt$_2$** and H_2 in CD2Cl2 at –20 °C.

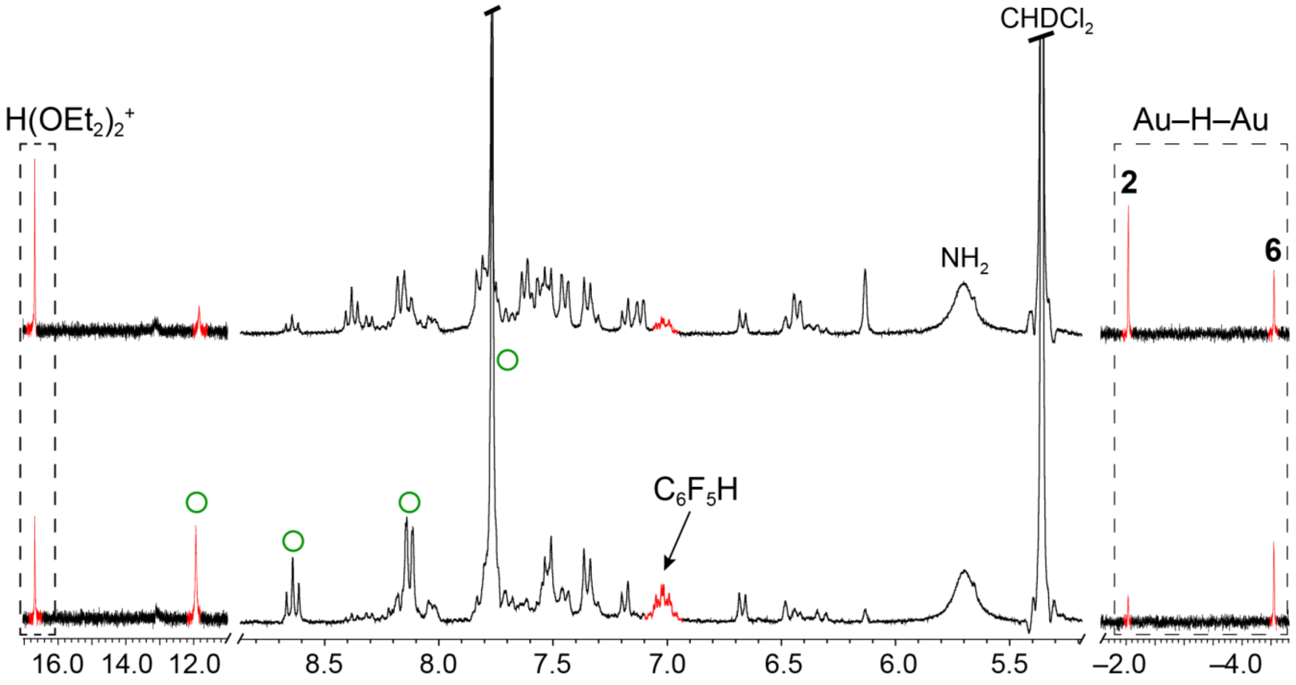

Figure S14. Evolution of the ^{1}H NMR spectrum of the mixture obtained after reacting **1·OEt$_2$** and H_2 in CD_2Cl_2 at –20 °C by exposing the sample to room temperature (5 x 2 minutes); green circles denote protonated 2,6-biarylpyridine ligand.

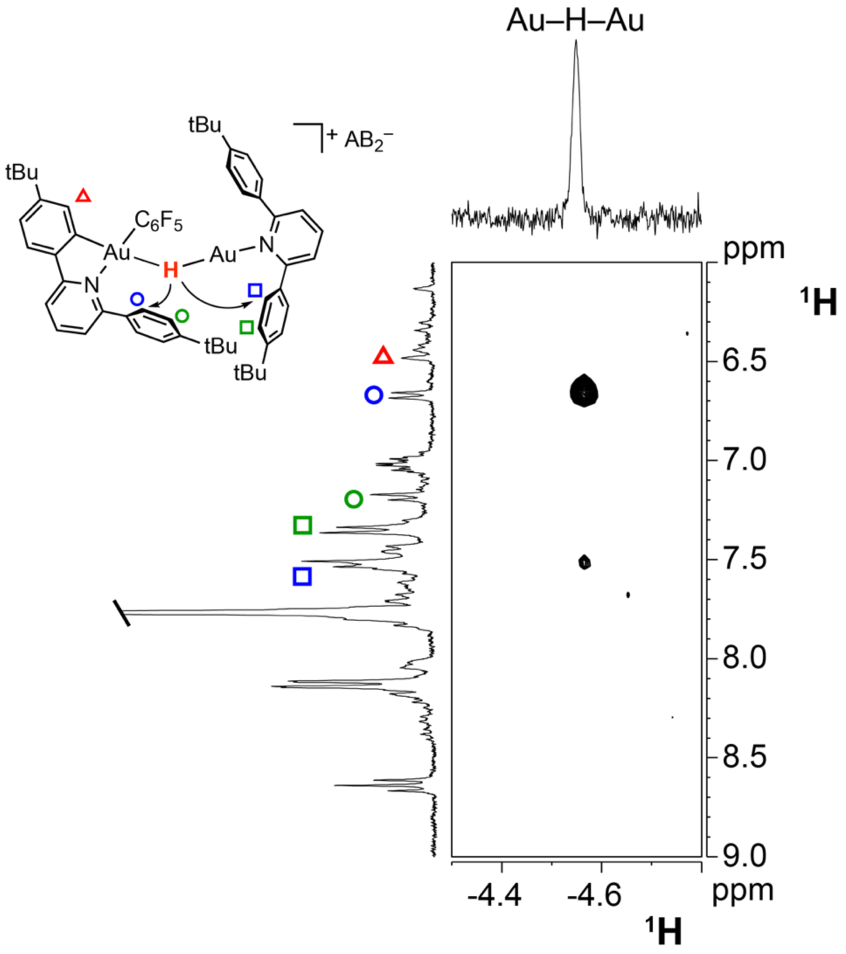

Figure S15. A section of the ^{1}H NOESY NMR spectrum of the mixture obtained after reacting **1·OEt₂** and H_2 in CD_2Cl_2 at –20 °C by exposing the sample to room temperature (5 x 2 minutes).

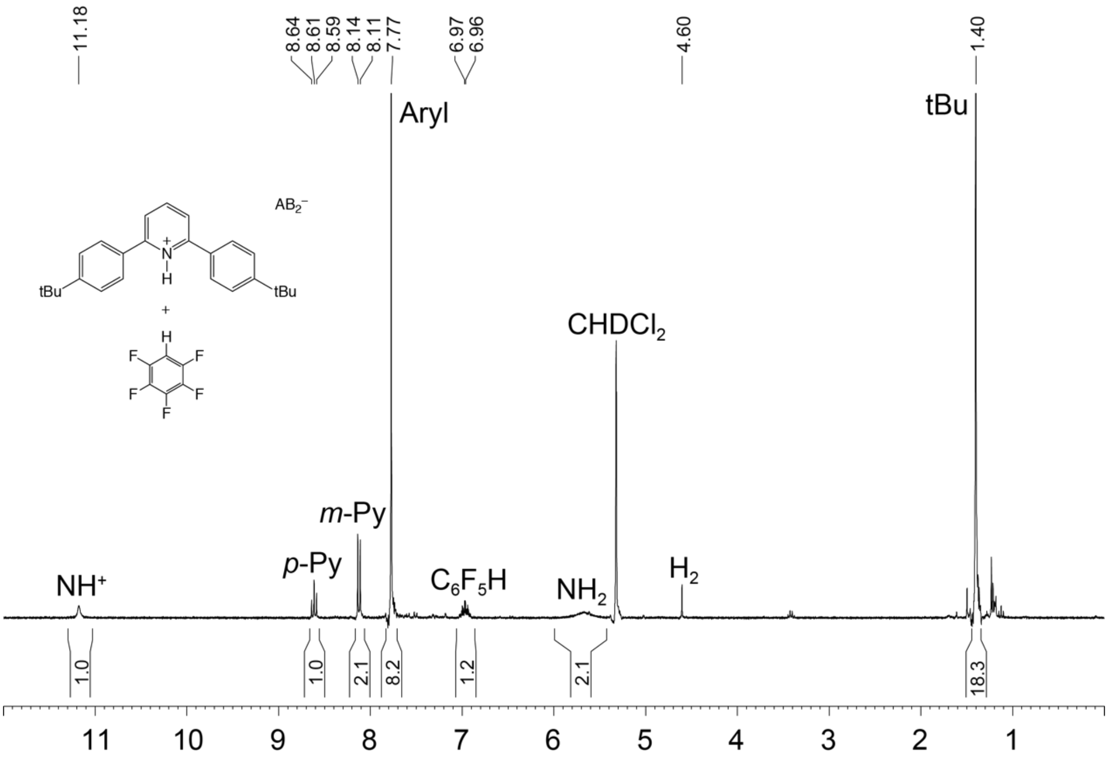

Figure S16. ^{1}H NMR spectrum obtained after the reaction between **1** and H_2 at room temperature.

5. Reactions with Hantzsch ester

Reaction in the presence of Et_2O:

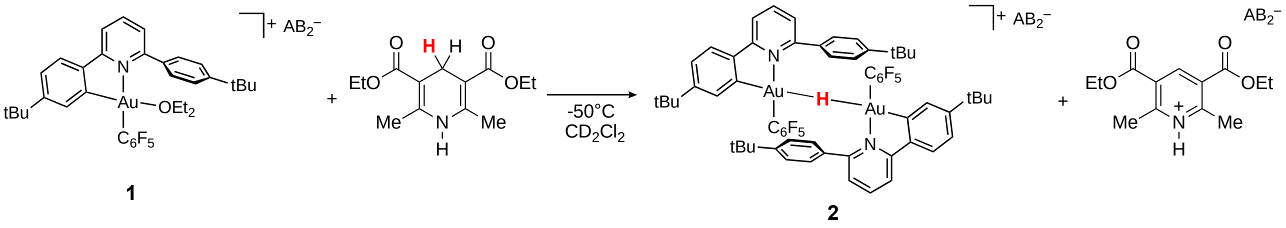

1·OEt$_2$ was generated at room temperature in the glovebox by reacting 5 mg of (C^N^C)AuC_6F_5 and 1 equivalent of $[H(OEt_2)_2][H_2N(B(C_6F_5)_3)_2]$ within a screw cap NMR tube in CD_2Cl_2. The NMR tube was then inserted into a cold bath at –78°C and a solution of diethyl 1,4-dihydro-2,6-dimethyl-3,5-pyridinedicarboxylate (2 equivalents in CD_2Cl_2) was injected through the septum of the NMR tube by a micrometric syringe. The solution was quickly shaken and inserted into the pre-cooled NMR probe and analyzed at -60°C. Quantitative conversion of **1** into **2** was observed instantaneously.

Reaction under base-free conditions:

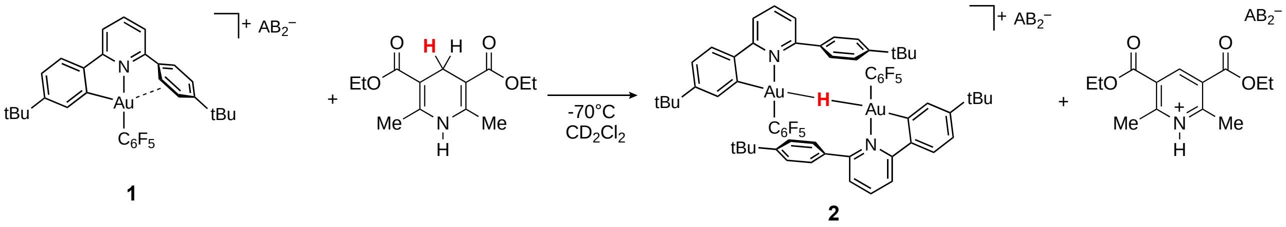

1·OEt$_2$ was generated at room temperature in the glovebox by reacting 5 mg of (C^N^C)AuC_6F_5 and 1 equivalent of $[H(OEt_2)_2][H_2N(B(C_6F_5)_3)_2]$ within a J-Young NMR tube in C_6D_5Cl. The tube was then dried under vacuum to remove any trace of Et_2O and redissolved in CD_2Cl_2. The resultant solution was transferred into a screw-cap NMR tube and inserted in a cold bath at -78°C. A solution containing 2 molar equivalents 1,4-dihydro-2,6-dimethyl-3,5-pyridinedicarboxylate was injected through the septum and the sample was quickly shaken before inserting the tube in the precooled NMR probe at -70°C.

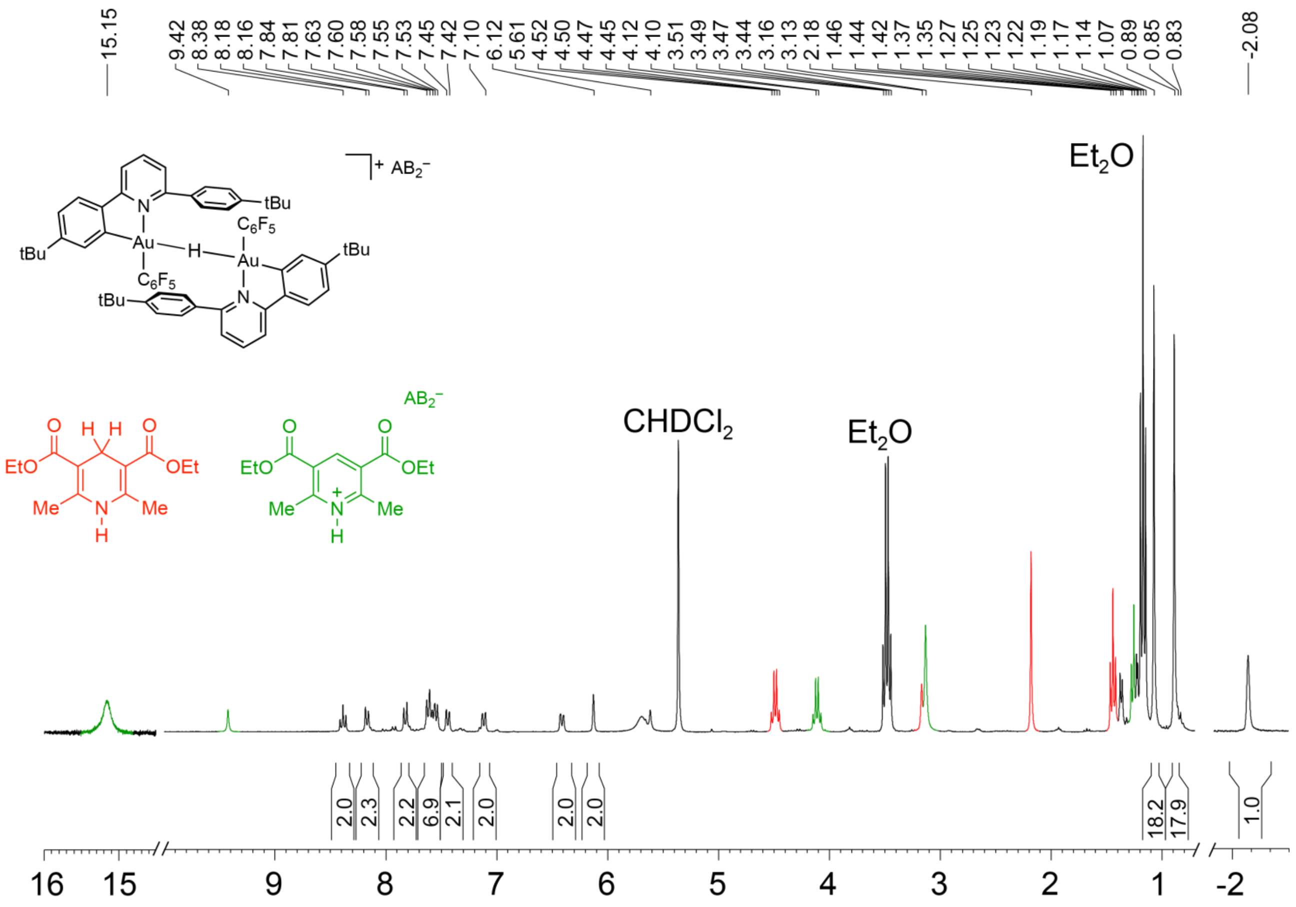

Figure S17. ^{1}H NMR spectrum obtained after the reaction between **1·OEt$_2$** and 1,4-dihydro-2,6-dimethyl-3,5-pyridinedicarboxylate at –50°C (CD_2Cl_2).

6. Reaction with 1,4-Dihydro-N-benzylnicotinamide

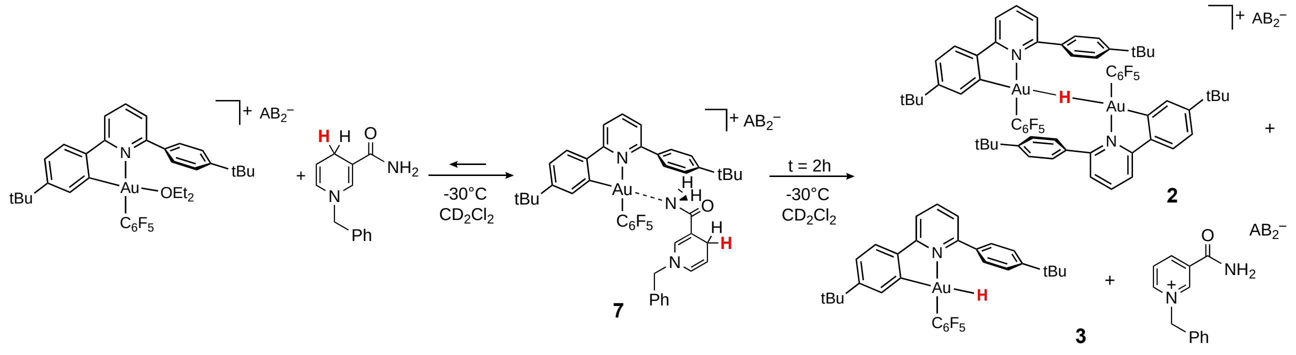

1·OEt$_2$ was generated at room temperature in the glovebox by reacting 5 mg of (C^N^C)AuC$_6$F$_5$ and 1 equivalent of $[H(OEt_2)_2][H_2N(B(C_6F_5)_3)_2]$ within a screw cap NMR tube in CD_2Cl_2. The NMR tube was then inserted into a cold bath at –78°C and a solution of diethyl 1,4-dihydro-N-benzylnicotinamide (1 equivalent in CD_2Cl_2) was injected through the septum of the NMR tube by a micrometric syringe. The solution was quickly shaken and inserted into the pre-cooled NMR probe and analyzed at -30°C. Data for **7**: ^{1}H NMR (300.13 MHz, CD_2Cl_2, 243 K, *J* values in Hz): 8.24 (t, $^3J_{HH}$=7.9, 1H, H1), 8.07 (d, $^3J_{HH}$=7.9, 1H, H2), 7.72 (d, $^3J_{HH}$=8.5, 1H, H5), 7.60 (t, $^3J_{HH}$=7.9, 1H, H2'), 7.52-7.38 (m, 8H, H5'+H6+H6+*m*-Ph+*p*-Ph), 7.22 (d, $^3J_{HH}$=7.2, 2H, *o*-Ph), 6.71 (s, 1H, H13), 6.51 (s, 1H, H8), 5.77 (d, 1H, $^3J_{HH}$=8.7,

H15), 5.69 (br s, 2H, NH_2 AB_2^-), 5.08 (br s, 2H, H10), 4.86 (br d, 1H, H14), 4.29 (s, 2H, H16), 2.66 (s, 2H, N–CH_2), 1.30 (s, 9H, CMe_3’), 1.15 ppm (s, overlapped with Et_2O, CMe_3).

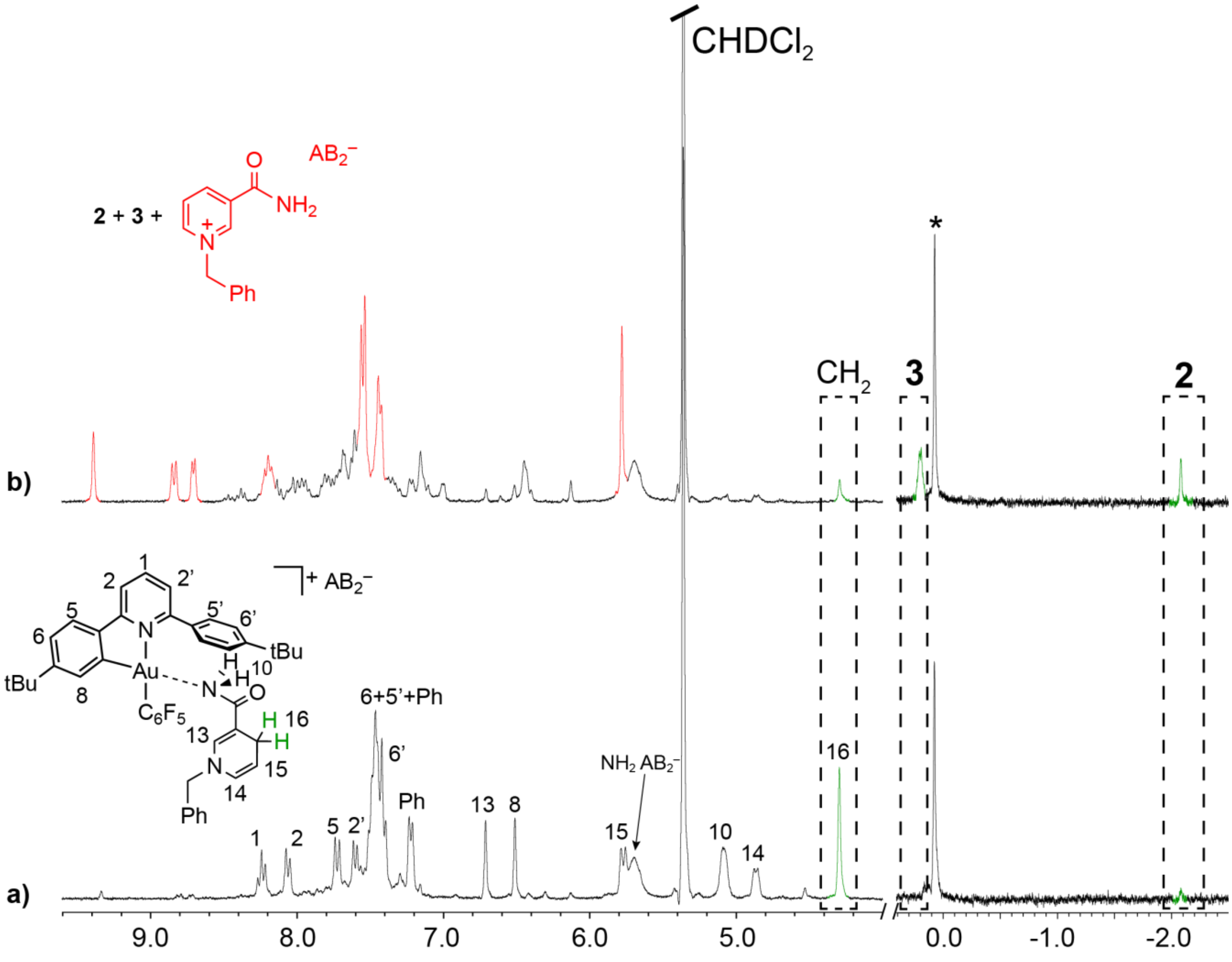

Figure S18. Evolution of the ^{1}H NMR spectrum of **7** (a, CD_2Cl_2, 243K) 30 minutes after mixing **1** and 1,4-dihydro-N-benzylnicotinamide (b).

7. Reaction with 1,4-cyclohexadiene

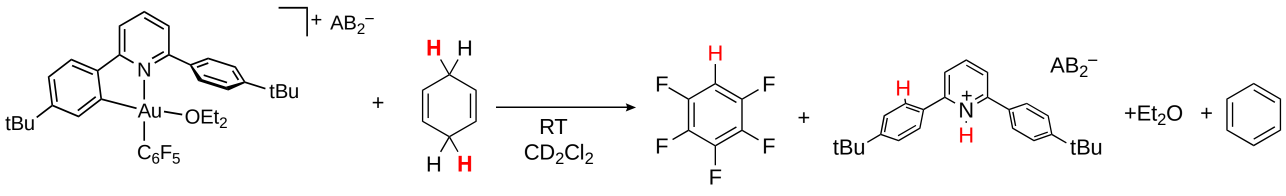

1

1·OEt₂ was generated at room temperature in the glovebox by reacting 5 mg of (C^N^C)AuC_6F_5 and 1 equivalent of $[H(OEt_2)_2][H_2N(B(C_6F_5)_3)_2]$ within a J-Young NMR tube in CD_2Cl_2 and mixed with 8 equivalents of 1,4-cyclohexadiene. The progress of the reaction was monitored by ^{1}H NMR over the period of two weeks.

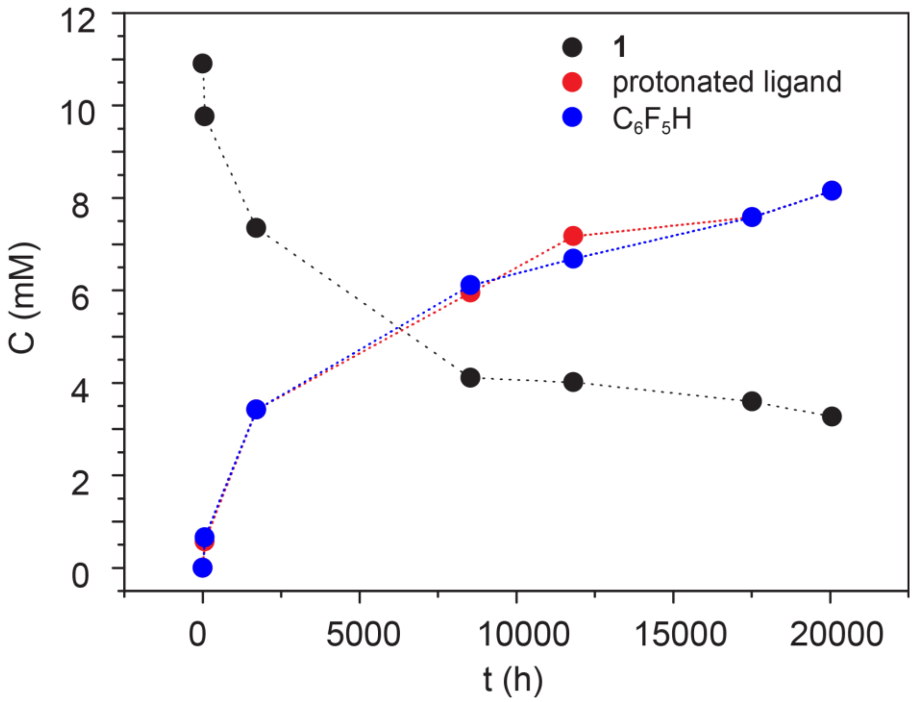

Figure S19. Kinetic profile for the reaction between **1** and 1,4-cyclohexadiene in CD_2Cl_2 at RT.

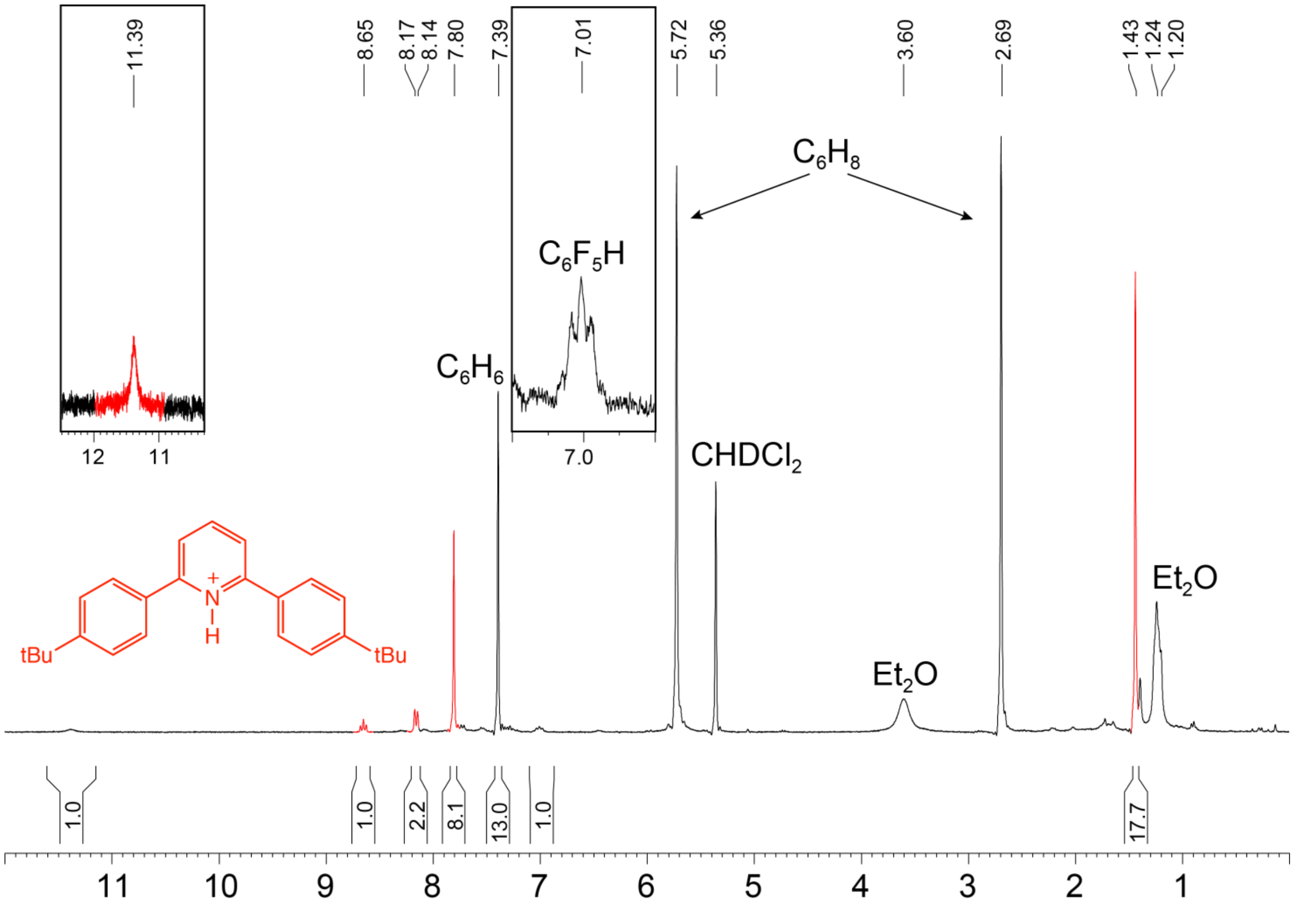

Figure S20. ^{1}H NMR spectrum obtained after 14 days from the mixing of **1·OEt$_2$** and 1,4-cyclohexadiene in CD_2Cl_2 at RT.

8. Reaction with cycloheptatriene

1·OEt$_2$ was generated at room temperature in the glovebox by reacting 7.5 mg of (C^N^C)AuC$_6$F$_5$ and 1 equivalent of [H(OEt$_2$)$_2$][H$_2$N(B(C$_6$F$_5$)$_3$)$_2$] within a Screw cap NMR tube in CD$_2$Cl$_2$. Successively, the sample was inserted in a cold bath at -78°C and 2 equivalents of cycloheptatriene were injected by using a microsyringe. The sample was then inserted in the pre-cooled NMR probe at –50 °C and the reaction was monitored by gradually increasing the temperature of the NMR probe.

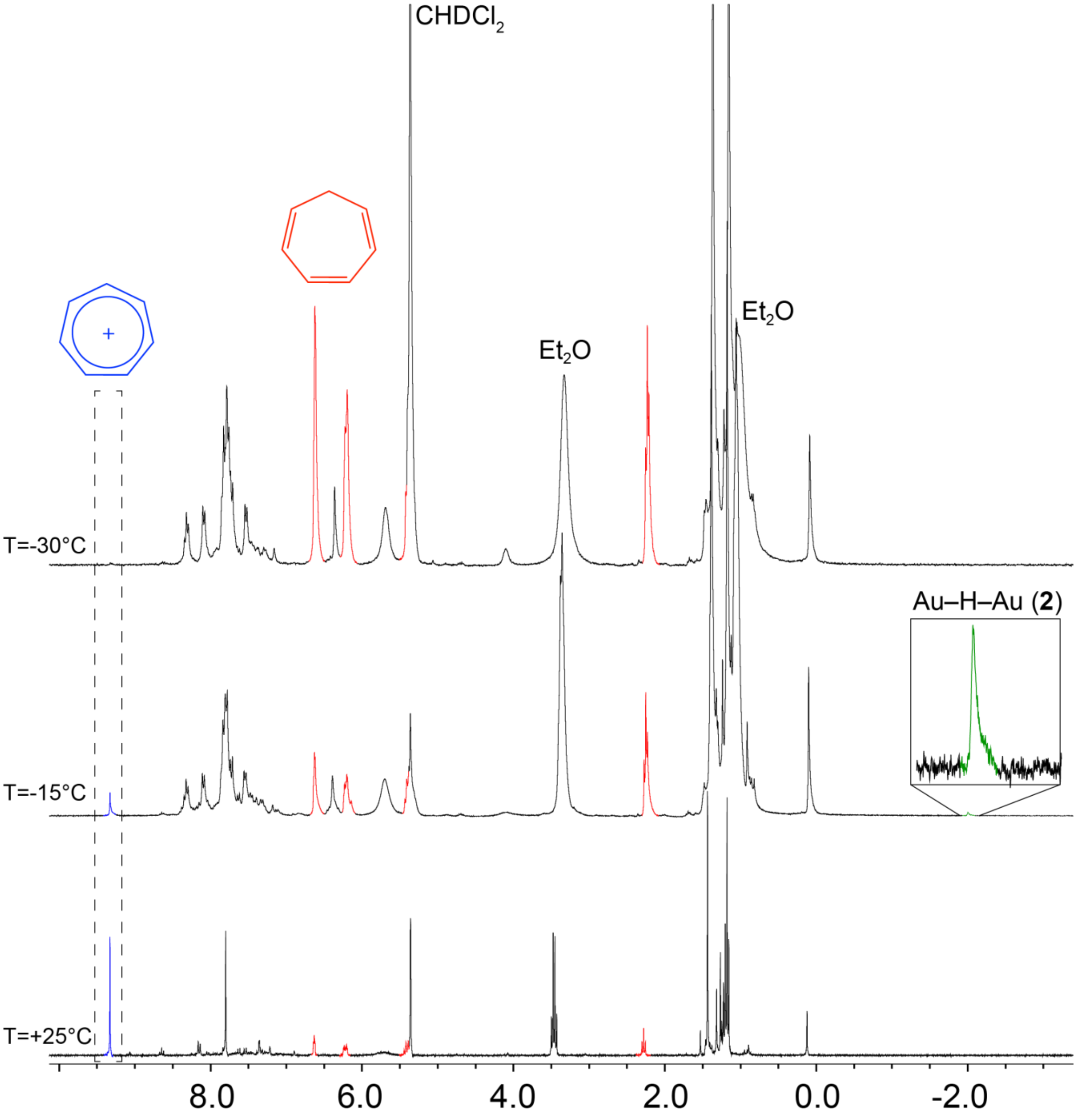

Figure S21. Thermal evolution of the ^{1}H NMR spectrum of **1·OEt$_2$** upon the reaction with 2 equivalents of cycloheptatriene in CD$_2$Cl$_2$.

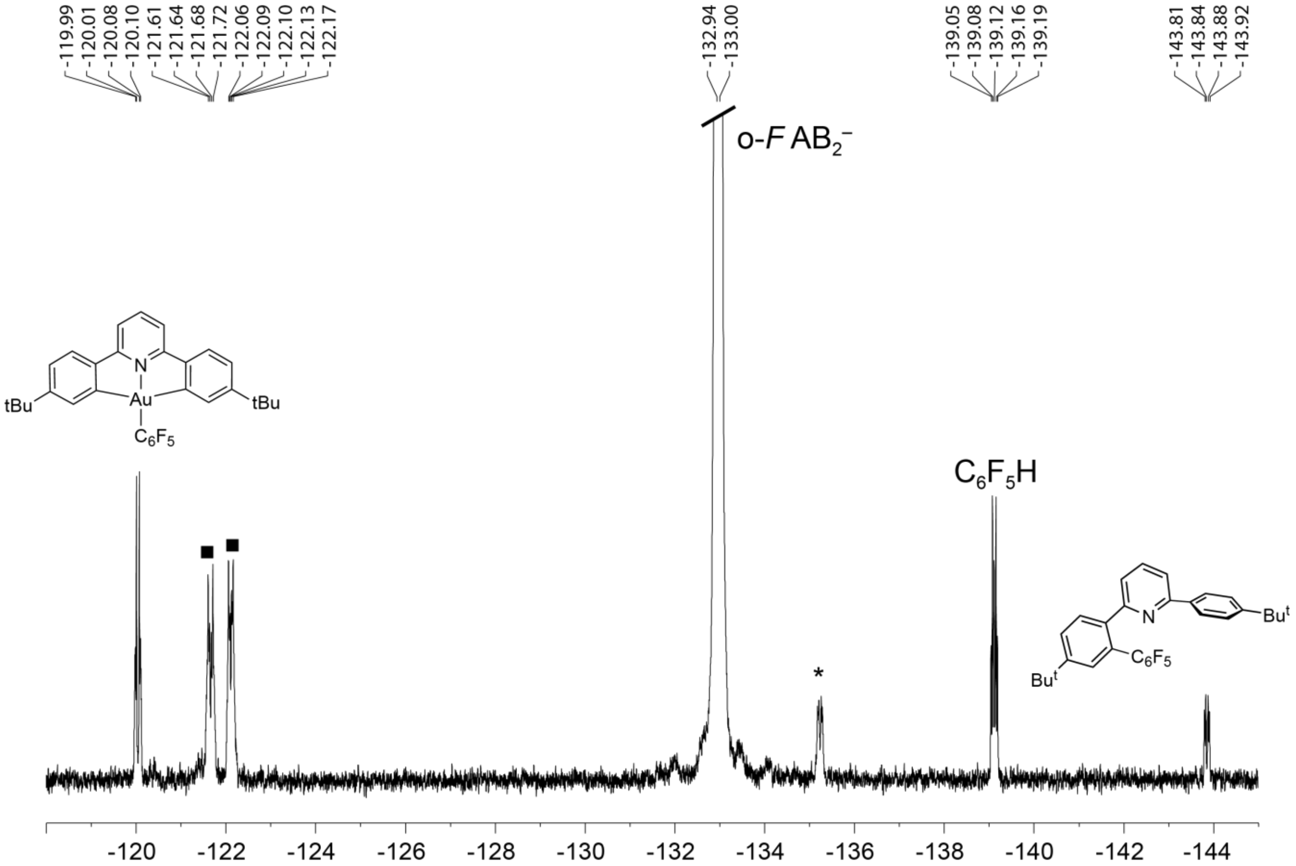

Figure S22. ^{19}F NMR spectrum of the reaction mixture obtaining by mixing **1·OEt$_2$** and 2 equivalents of cycloheptatriene in CD_2Cl_2 (T=25°C); squares denote unidentified Au(III) side products, asterisk denote inert minor impurity of AB_2^-.

9. Computational.

Computational studies were performed for model systems lacking the *t*Bu substituents at the Au-bound phenyl rings. Me_3SiH was used as a model for Et_3SiH. All calculations were done using Gaussian 09.[S3] Structures were optimized at the B3LYP[S4-S6]/def2-SVP[S7] level (with a corresponding ECP at Au[S8]) for the gas phase. The nature of stationary points was checked by vibrational analyses. Improved single-point energies were obtained with M06[S9]/cc-pVTZ[S10-S13] (and using the corresponding ECP at Au[S14,S15]) including a PCM(CH_2Cl_2) solvent correction.[S16-S18] These were combined with the thermal corrections (enthalpy and entropy) at 250 K, 1 bar, obtained from the B3LYP/def2-SVP vibrational analyses. Entropy contributions to the free energy were scaled by a factor of 0.67 to account for reduced freedom in solution.[S19,S20]

Table S1. Total and relative energies for species studied.[a]

**Name**	**Formula**	***H*corr 250 K**	***TS*corr 250 K**	***E*elec**	***G***	**on scale**	***G*rel kcal/mol**
LAuAr_+	C23H12AuF5N	0.30953	0.06361	-1572.76947	-1572.50256	-1572.50256	(0)
LAuAr_OMe2_+	C25H18AuF5NO	0.39624	0.07072	-1727.77613	-1727.42728	-1572.51668	-8.86
LAuAr_H_transC	C23H13AuF5N	0.31696	0.06463	-1573.53618	-1573.26252	-1573.26252	(0)
LAuAr_H_cisC	C23H13AuF5N	0.31729	0.06359	-1573.54762	-1573.27293	-1573.27293	-6.53
dimer1_HtransC+	C46H25Au2F10N2	0.62781	0.10693	-3146.35102	-3145.79485	-1573.29229	-18.68
dimer2_HtransC+	C46H25Au2F10N2	0.62809	0.10591	-3146.35174	-3145.79461	-1573.29204	-18.53
dimer1_HtransN+	C46H25Au2F10N2	0.62838	0.10636	-3146.31358	-3145.75646	-1573.25390	5.41
dimer2_HtransN+	C46H25Au2F10N2	0.62833	0.10763	-3146.31314	-3145.75693	-1573.25437	5.12
**H2**							
H2	H2	0.01263	0.01259	-1.17079	-1.16660		
OMe2	C2H6O	0.08336	0.02415	-154.97778	-154.91060		
OMe2_H+	C2H7O	0.09709	0.02542	-155.37210	-155.29204	-310.20264	
OMe2_2_H+	C4H13O2	0.17968	0.03687	-310.37865	-310.22367	-310.22367	-13.20
Au(+) + H2 + OMe2						***-1728.57977***	(0)
LAuAr_H2_+	C23H14AuF5N	0.32572	0.06408	-1573.94205	-1573.65926	-1728.56986	6.21
LAuAr_H2_+OMe2_transTS	C25H20AuF5NO	0.41052	0.07395	-1728.92525	-1728.56428	-1728.56428	9.72
LAuArH_HOMe2_+	C25H20AuF5NO	0.41446	0.07344	-1728.92827	-1728.56301	-1728.56301	10.51
AuH + HOMe2(+)						-1728.55456	15.82
Au(+) + H2 + 2 OMe2						***-1883.49037***	(0)
LAuAr_H2_+	C23H14AuF5N	0.32572	0.06408	-1573.94205	-1573.65926	-1883.48047	6.21
LAuAr_H2_+OMe2_transTS	C25H20AuF5NO	0.41052	0.07395	-1728.92525	-1728.56428	-1883.47488	9.72
LAuArH_HOMe2_+	C25H20AuF5NO	0.41446	0.07344	-1728.92827	-1728.56301	-1883.47362	10.51
AuH + H[OMe2]2(+)						-1883.48620	2.62
**HSiMe3**							
HSiMe3	C3H10Si	0.12410	0.03051	-409.79628	-409.69262		
SiMe3_+	C3H9Si	0.11543	0.03136	-408.99189	-408.89748		
SiMe3_+_OMe2	C5H15OSi	0.20326	0.03865	-564.03202	-563.85466		
Au(+) + HSiMe3						-1982.19519	
AuH + Me3Si(+)						-1982.16000	22.08
Au(+) + HSiMe3 + OMe2						***-2137.10579***	(0)
LAuAr_HSiMe3_+	C26H22AuF5NSi	0.43570	0.07670	-1982.58791	-1982.20360	-2137.11421	-5.28

**Name**	**Formula**	***H*corr 250 K**	***TS*corr 250 K**	***E*elec**	***G***	**on scale**	**Grel kcal/mol**
LAuAr_HSiMe3_+OMe2_transTS	C28H28AuF5NOSi	0.52117	0.08304	-2137.57270	-2137.10718	-2137.10718	-0.87
AuH + Me3SiOMe2(+)						-2137.11718	-7.15
**HBPin**							
HBPin	C6H13BO2	0.19684	0.03338	-411.74078	-411.56630		
BPin+	C6H12BO2	0.18594	0.03369	-410.86753	-410.70417		
BPin+_OMe2	C8H18BO3	0.27520	0.04241	-565.97125	-565.72447		
Au(+) + HBPin						-1984.06886	
AuH + BPin(+)						-1983.96669	64.11
Au(+) + HBPin + OMe2						***-2138.97946***	(0)
LAuAr_HBPin_+	C29H25AuBF5NO2	0.50879	0.08088	-1984.52461	-1984.07001	-2138.98062	-0.72
LAuAr_HBPinOMe2_+	C31H31AuBF5NO3	0.59356	0.08604	-2139.51810	-2138.98219	-2138.98219	-1.71
AuH + Me2OBPin(+)						***-2138.98699***	-4.72
**PyCar**							
PyCarH	C11H15NO4	0.26520	0.04931	-783.64280	-783.41064		
PyCar+	C11H14NO4	0.25568	0.04818	-782.88613	-782.66274		
Au(+) + PyCarH						***-2355.91320***	(0)
LAuAr_+_PyCar_TS	C34H27AuF5N2O4	0.57195	0.09173	-2356.41783	-2355.90735	-2355.90735	3.67
AuH + PyCar(+)						-2355.92526	-7.57
**Ph3CH**							
Ph3CH	C19H16	0.30230	0.04678	-733.30709	-733.03614		
Ph3C+	C19H15	0.29160	0.04435	-732.50741	-732.24552		
Au(+) + Ph3CH						-2305.53870	
AuH + Ph3C(+)						-2305.50805	19.24
**CHDH**							
CHDH	C6H8	0.12616	0.02654	-233.30295	-233.19457		
CHD+	C6H7	0.11467	0.02663	-232.50622	-232.40938		
Au(+) + CHDH						***-1805.69713***	(0)
LAuAr_+_CHDH_react	C29H20AuF5N	0.43755	0.07880	-1806.07574	-1805.69098	-1805.69098	3.86
LAuAr_+_CHDH_TS	C29H20AuF5N	0.43255	0.07293	-1806.05498	-1805.67128	-1805.67128	16.22
LAuArH_CHD+_prod	C29H20AuF5N	0.43335	0.07670	-1806.05418	-1805.67222	-1805.67222	15.63
AuH + CHD(+)						-1805.67190	15.83
**BnH**							
BnH	C7H8	0.13288	0.03110	-271.42979	-271.31775		
Bn+	C7H7	0.12209	0.02784	-270.58824	-270.48480		
Au(+) + BnH						-1843.82031	
AuH +Bn(+)						-1843.74733	45.80

[a] Chosen reference values in bold. Free energies calculated as $G = E\mathrm{elec} + H\mathrm{corr} - 0.67\ TS\mathrm{corr}$.

References

S3. *Gaussian 09 B.01*: M. J. Frisch, G. W. Trucks, H. B. Schlegel, G. E. Scuseria, J. R. Cheeseman, G. Scalmani, V. Barone, B. Mennucci, G. A. Petersson, H. Nakatsuji, M. Caricato, X. Li, H. P. Hratchian, A. F. Izmaylov, J. Bloino, G. Zheng, J. L. Sonnenberg, M. Hada, M. Ehara, K. Toyota, R. Fukuda, J. Hasegawa, M. Ishida, T. Nakajima, Y. Honda, O. Kitao, H. Nakai, T. Vreven, J. A. J. Montgomery, J. E. Peralta, F.

Ogliaro, M. Bearpark, J. J. Heyd, E. Brothers, K. N. Kudin, V. N. Staroverov, R. Kobayashi, J. Normand, K. Raghavachari, A. Rendell, J. C. Burant, S. S. Iyengar, J. Tomasi, M. Cossi, N. Rega, J. M. Millam, M. Klene, J. E. Knox, J. B. Cross, V. Bakken, C. Adamo, J. Jaramillo, R. Gomperts, R. E. Stratmann, O. Yazyev, A. J. Austin, R. Cammi, C. Pomelli, J. W. Ochterski, R. L. Martin, K. Morukuma, V. G. Zakrzewski, G. A. Voth, P. Salvador, J. J. Dannenberg, S. Dapprich, A. D. Daniels, O. Farkas, J. B. Foresman, J. V. Ortiz, J. Ciolowski and D. J. Fox, Gaussian, Inc., Wallingford CT, 2009

S4. A. D. Becke, *J. Chem. Phys.*, 1993, **98**, 5648-5652.

S5. A. D. Becke, *J. Chem. Phys.*, 1993, **98**, 1372-1377.

S6. C. T. Lee, W. T. Yang and R. G. Parr, *Phys. Rev. B*, 1988, **37**, 785-789.

S7. F. Weigend and R. Ahlrichs, *Phys. Chem. Chem. Phys.*, 2005, **7**, 3297-3305.

S8. D. Andrae, U. Haussermann, M. Dolg, H. Stoll and H. Preuss, *Theor. Chim. Acta*, 1990, **77**, 123-141.

S9. Y. Zhao and D. G. Truhlar, *Theor. Chem. Acc.*, 2008, **120**, 215-241.

S10. T. H. Dunning, *J. Chem. Phys.*, 1989, **90**, 1007-1023.

S11. D. E. Woon and T. H. Dunning, *J. Chem. Phys.*, 1993, **98**, 1358-1371.

S12. K. L. Schuchardt, B. T. Didier, T. Elsethagen, L. S. Sun, V. Gurumoorthi, J. Chase, J. Li and T. L. Windus, *J. Chem. Inf. Model.*, 2007, **47**, 1045-1052.

S13. D. Feller, *J. Comput. Chem.*, 1996, **17**, 1571-1586.

S14. D. Figgen, G. Rauhut, M. Dolg and H. Stoll, *Chem. Phys.*, 2005, **311**, 227-244.

S15. K. A. Peterson and C. Puzzarini, *Theor. Chem. Acc.*, 2005, **114**, 283-296.

S16. S. Miertus and J. Tomasi, *Chem. Phys.*, 1982, **65**, 239-245.

S17. S. Miertus, E. Scrocco and J. Tomasi, *Chem. Phys.*, 1981, **55**, 117-129.
